# From Motion Artifacts to Clinical Insight: Multi-Modal Deep Learning for Robust Arrhythmia Screening in Ambulatory ECG Monitoring

**DOI:** 10.3390/s26041135

**Published:** 2026-02-10

**Authors:** Pierre Boulanger

**Affiliations:** Department of Computing Science, University of Alberta, Edmonton, AB T6G 2R3, Canada; pierreb@ualberta.ca

**Keywords:** arrhythmia detection, motion artifacts, sensor fusion, wearable ECG, deep learning, false alarm reduction, MIT-BIH, accelerometer

## Abstract

Motion artifacts corrupt wearable ECG signals and generate false alarms of arrhythmias, limiting the clinical adoption of continuous cardiac monitoring. We present a dual-stream deep learning framework for motion-robust binary arrhythmia classification through multi-modal sensor fusion and multi-SNR training. ResNet-18 processes ECG spectrograms, while CNN-BiLSTM encodes accelerometer motion patterns; attention-gated fusion with gate diversity regularization adaptively weights modalities based on signal reliability. Training in MIT-BIH data augmented at three noise levels (24, 12, 6 dB) enables noise-invariant learning with successful generalization to unseen conditions. The framework achieves 99.5% accuracy under clean signals, gracefully degrading to 88.2% at extreme noise (−6 dB SNR)—a 46% improvement over training with single-SNR. The high gate diversity (σ>0.37) confirms adaptive context-dependent fusion. With a 0.09% false positive rate and real-time processing (238 beats/second), the system provides practical continuous arrhythmia screening, establishing the foundation for hierarchical monitoring systems where binary screening activates detailed multi-class diagnosis.

## 1. Introduction

Cardiac arrhythmias cause approximately 60,000 cardiac arrests per year in Canada, with atrial fibrillation (AF) affecting more than 33 million people around the world and increasing the risk of stroke five times [1]. Wearable electrocardiogram (ECG) monitors offer unprecedented opportunities for continuous arrhythmia surveillance in ambulatory settings, but a critical limitation undermines their clinical adoption: motion artifacts generate false alarms that overwhelm healthcare providers and undermine patient trust [2]. Studies of intensive care unit (ICU) alarms reveal that 85–99% of arrhythmia alerts are false positives, primarily caused by patient movement, electrode displacement, and electromyographic interference [3,4]. This false alarm rate causes “alarm fatigue,” where healthcare providers become desensitized to alerts and potentially miss real-life-threatening events [5].

Traditional ECG processing algorithms employ signal quality indices (SQI) that attempt to identify corrupted segments and suppress analysis during poor-quality intervals [6]. However, SQI-based approaches face an inherent dilemma: aggressive quality thresholds reduce false positives but risk missing genuine arrhythmia that occurs during movement. Recent deep learning approaches achieve high accuracy on benchmark databases with clean signals [7,8], but performance degrades substantially on motion-corrupted data.

We address these challenges through attention-gated multi-modal fusion, integrating ECG spectrogram analysis with tri-axial accelerometer measurements that quantify patient movement. Our framework processes ECG and accelerometer data through specialized parallel encoders: ResNet-18 extracts 512-dimensional features from ECG spectrograms, while CNN-BiLSTM networks extract 128-dimensional motion features from accelerometer time-series. Learnable attention gates dynamically balance the contribution of each modality:

(1) $$f_{\mathrm{fused}}=\left[g_{\mathrm{ECG}}⊙f_{\mathrm{ECG}};\;g_{\mathrm{ACC}}⊙f_{\mathrm{ACC}}\right]$$

where $g_{\mathrm{ECG}},g_{\mathrm{ACC}}\in \left[0,1\right]$ are computed using small multilayer perceptrons. Regularization of the diversity of the gates prevents the collapse to constant weights. During training on multi-SNR augmented data (24, 12, 6 dB), the network learns an ECG-dominant fusion policy ($g_{\mathrm{ECG}}\approx 0.71$, $g_{\mathrm{ACC}}\approx 0.41$) with substantial sample-to-sample variation (σ(g)>0.37), that confirms adaptive context-dependent fusion.

The remainder of this paper is organized as follows. Section 2 reviews related work on motion artifact rejection, deep learning for arrhythmia detection, signal quality assessment, and multimodal fusion. Section 3 presents our methodology, including signal processing, network architecture, and training strategy. Section 4 describes the deep learning architecture in detail. Section 5 presents experimental results across synthetic noise conditions and real-world activity validation. Section 6 discusses the hierarchical two-stage classification system and its deployment considerations. In Section 7 we conclude with a summary of contributions and directions for future work.

## 2. Related Work

Motion artifact rejection in ambulatory ECG monitoring has traditionally relied on classical signal processing techniques. Adaptive filtering approaches, such as those developed by Pan and Tompkins [9], provide effective removal of baseline wander and high-frequency noise, while wavelet-based methods [6] enable multi-resolution decomposition that can isolate different artifact components. Independent Component Analysis (ICA) has shown promise for blind source separation when multiple observations are available. However, these classical methods face fundamental limitations when artifacts exhibit spectral overlap with cardiac signals, as the frequency content of motion-induced disturbances often coincides with diagnostically relevant ECG features. Furthermore, single-lead wearable devices cannot provide the multiple independent observations required for ICA-based separation, limiting the applicability of these multi-channel techniques in practical ambulatory scenarios.

The advent of deep learning has revolutionized the detection of arrhythmias in ECG signals, with numerous architectures that demonstrate performance that is approaching or exceeding the level of human experts. Rajpurkar et al. [7] achieved cardiology-level performance with an F1 score of 0.83 using a 34-layer convolutional neural network trained on a large clinical dataset. Building on this foundation, Hannun et al. [8] demonstrated 97.7% precision using deeper residual network architectures that allow the training of very deep models through skip connections. Subsequent work has explored attention mechanisms [10] that allow models to focus on diagnostically relevant temporal regions, as well as transformer architectures [11,12] originally developed for natural language processing but adapted for sequence modeling of cardiac signals. Transfer learning approaches [13,14] have proven particularly valuable in enabling knowledge transfer across datasets with different acquisition protocols or patient populations. The medical community has increasingly recognized AI-enhanced ECG analysis as transformative for the management of cardiovascular disease [15,16], with studies that demonstrate capabilities ranging from the detection of subtle arrhythmias to the prediction of future cardiac events. Ansari et al. [17] provided a comprehensive review documenting the remarkable progress in deep learning for the detection of ECG arrhythmias from 2017 to 2023. Despite these impressive achievements, a critical limitation remains largely unaddressed: these results were obtained primarily on curated datasets with minimal motion artifacts, and model performance degrades substantially when evaluated on motion-corrupted data representative of real-world ambulatory conditions.

In addition to artifact removal and robust classification, signal quality assessment provides a framework for quantifying ECG corruption and identifying segments suitable for diagnostic analysis. Signal quality indices (SQIs) employ metrics such as baseline drift magnitude, high-frequency noise power, and frequency consistency of waveform morphology [18] to estimate the reliability of recorded signals. The PhysioNet/Computing in Cardiology Challenge 2015 [19] specifically addressed the reduction of false alarms in the monitoring of arrhythmias in the intensive care unit, promoting the development of sophisticated signal selection algorithms and machine learning methods for alarm suppression based on quality metrics. Although SQI approaches allow intelligent rejection of corrupted segments and prevent analysis of unreliable data, they cannot recover diagnostic information from degraded signals or reliably distinguish genuine arrhythmias from motion-induced artifacts that mimic pathological patterns.

Multimodal sensor fusion represents an alternative paradigm that uses complementary information from multiple synchronized sensors rather than relying solely on ECG quality assessment or artifact removal. Accelerometer-based approaches [20,21] have demonstrated substantial improvements in heart rate estimation during exercise by providing direct measurement of body motion that can inform the detection and compensation of artifacts. Castanedo [22] surveyed sensor fusion techniques in detail, categorizing approaches into early fusion (integration of features at the start of the classification), late fusion (decision-level combination after independent classification) and hybrid fusion strategies that combine both paradigms. These categorizations provide a useful framework for understanding the design space of multimodal systems, although the optimal fusion strategy remains application-dependent.

The development and validation of these diverse approaches have been enabled by public benchmark databases that provide standardized evaluation frameworks for reproducible research. PhysioNet has established itself as the leading resource for physiological signal databases [18,23], hosting datasets ranging from the MIT-BIH foundational ventricular dysfunction Database to modern large-scale collections. Recent additions include the PTB-XL database [24] that contains 21,837 clinical ECGs recordings with comprehensive diagnostic labels, and the Chapman–Shaoxing database [25] that provides 10 s ECGs from 45,152 patients. The Cardiology PhysioNet/Computer Challenge series has proven particularly influential by focusing research attention on specific clinically relevant problems through competitive evaluation, including the 2015 challenge [19] on false alarm reduction in intensive care monitoring and the 2020–2021 challenges [26] on multi-label ECG classification in diverse datasets. These standardized benchmarks enable direct performance comparison between methods and drive systematic progress in well-defined clinical objectives, although the gap between benchmark performance and real-world deployment remains substantial due to differences in data quality, patient populations, and operational constraints.

### Gaps Addressed by This Work

Despite substantial progress in deep learning for arrhythmia detection, critical gaps remain that limit translation from research to clinical deployment. Most studies report accuracy exclusively on clean benchmark datasets without systematic evaluation at controlled noise levels, leaving motion robustness largely uncharacterized and preventing meaningful cross-method comparison. Closely related, few studies measure false alarm rates in healthy subjects performing everyday activities, despite false positives being the primary clinical problem that limits the adoption of wearable monitors; the literature focuses predominantly on sensitivity to detect arrhythmias, while specificity during normal sinus rhythm under motion corruption receives minimal attention. These evaluation gaps are compounded by the single-modal limitation inherent in pure ECG analysis, since high-performance algorithms that rely exclusively on cardiac electrical signals lack the contextual information necessary to distinguish artifact-induced waveform abnormalities from genuine pathology. Finally, algorithms validated exclusively on stationary recordings from hospitalized patients may fail to generalize to ambulatory settings characterized by substantial and unpredictable motion, as the distribution shift encompasses not only motion artifacts but also electrode placement variability, skin impedance changes, and different activity contexts absent from inpatient datasets. The MIT-BIH Arrhythmia Database, augmented with controlled synthetic noise at six SNR levels (24, 18, 12, 6, 0, −6 dB), was used to systematically characterize performance degradation under increasing corruption, and the ScientISST MOVE database, providing validation of genuine motion artifacts from healthy subjects performing everyday activities (baseline, greetings, gesticulate, walking, running), was used to enable direct measurement of false positive rates throughout the activity spectrum.

## 3. Mathematical Framework

### 3.1. Problem Formulation

Let $x_{\mathrm{ECG}}\left[n\right]$ denote a single-lead ECG signal and $a\left[n\right]={\left[a_{x}\left[n\right],a_{y}\left[n\right],a_{z}\left[n\right]\right]}^{T}$ represent tri-axial accelerometer measurements, where *n* indexes discrete time samples in $f_{s}=1000$ Hz. The goal is to estimate the posterior probability $P(C|x_{\mathrm{ECG}},a)$ of the cardiac state C∈{Normal,Arrythmia} given both sensor modalities. See Table 1 for a definition of both classes.

Under motion artifacts, the observed ECG is as follows:

(2) $$x_{\mathrm{ECG}}\left[n\right]=s_{\mathrm{cardiac}}\left[n\right]+s_{\mathrm{artifact}}\left[n\right]+\eta \left[n\right]$$

where $s_{\mathrm{cardiac}}\left[n\right]$ represents the true cardiac signal, $s_{\mathrm{artifact}}\left[n\right]$ denotes motion-induced corruption correlated with a[n], and η[n] is additive white noise.

The key challenge in ambulatory cardiac monitoring is that conventional arrhythmia detectors operating solely on the corrupted ECG signal $x_{\mathrm{ECG}}\left[n\right]$ cannot distinguish between genuine pathological rhythm disturbances and artifact-induced waveform distortions $s_{\mathrm{artifact}}\left[n\right]$ that mimic the morphology of the arrhythmia. Our multi-modal approach addresses this fundamental ambiguity by leveraging synchronized tri-axial accelerometer measurements, a[n], to estimate instantaneous motion intensity and modulate arrhythmia detection accordingly, allowing the fusion network to suppress false alarms during periods of elevated body motion while maintaining sensitivity for genuine cardiac events.

### 3.2. Signal Preprocessing

#### 3.2.1. Bandpass Filtering

Both the ECG and accelerometer signals are filtered by a fourth-order Butterworth bandpass filter [28]. Raw ECG signals x[n] sampled at $f_{s}=360$ Hz undergo bandpass filtering to remove physiologically irrelevant frequency components:

(3) $$x_{\mathrm{filtered}}\left[n\right]=H_{\mathrm{BP}}\left(z\right)*x\left[n\right]$$

where $H_{\mathrm{BP}}\left(z\right)$ is a fourth-order Butterworth bandpass filter with:

- Lower cutoff: $f_{\mathrm{low}}$ Hz (removes baseline wander, respiration artifacts);
- Upper cutoff: $f_{\mathrm{high}}$ Hz (removes powerline noise and EMG artifacts);
- Passband: [0.5,50] Hz (preserves the P-wave, QRS complex, T-wave);
- Filter order: 4 (provides −80 dB attenuation at 0.1 Hz and 120 Hz);
- Implementation: Cascaded second-order sections for numerical stability.

The Butterworth design ensures a maximally flat passband response, avoiding ripples that could distort cardiac waveform morphology critical for arrhythmia classification. The filter provides −40 dB/decade rolloff outside the passband with attenuation of −3 dB at cutoff frequencies. For ECG, the cutoff frequencies are $f_{\mathrm{low}}=0.5$ Hz and $f_{\mathrm{high}}=40$ Hz to preserve the QRS complexes while attenuating the baseline wander and high-frequency noise. For the accelerometer signal, the filter parameters are set to $f_{\mathrm{low}}=0.1$ Hz and $f_{\mathrm{high}}=20$ Hz to capture human motion frequencies while rejecting DC drift and high-frequency vibrations.

#### 3.2.2. Normalization

Both the ECG and accelerometer signals undergo Z-score normalization to ensure consistent scaling between subjects and recordings, applying separately to each modality according to:

(4) $$\tilde{x}\left[n\right]=\frac{x\left[n\right]-\mu_{w}}{\sigma_{w}}$$

where $\mu_{w}$ and $\sigma_{w}$ represent the mean and standard deviation computed on a 10 s sliding window centered on the sample *n*. This windowed approach, rather than global normalization, provides critical advantages for ambulatory analysis by adapting to amplitude variations caused by physiological factors such as electrode impedance changes, respiration-induced baseline wander, and postural shifts, while preserving relative morphology within each cardiac cycle—the P-wave, QRS complex, and T-wave amplitudes maintain their proportional relationships that encode crucial diagnostic information about conduction patterns. The 10 s window duration (approximately 10–15 heartbeats at typical resting rates) provides sufficient statistical samples for stable estimates while remaining short enough to track dynamic changes during physical activity transitions. For edge cases where the sliding window extends beyond signal boundaries, we employ symmetric padding to prevent artificial discontinuities, and apply a minimum variance threshold of $\sigma_{\min}=0.01$ when encountering flat-line segments to prevent numerical instability. This windowed normalization strategy mirrors preprocessing in clinical Holter analysis systems, allowing the neural network to focus on diagnostically relevant temporal patterns and morphological features rather than learning to compensate for amplitude scaling artifacts, with the accelerometer signal normalized independently across all three axes before computing magnitude ∥a[n]∥ to ensure orientation-independent motion intensity estimates.

### 3.3. ECG Spectrogram Analysis

The Short-Time Fourier Transform (STFT) decomposes the ECG into a time-frequency representation:

(5) $$X\left[m,k\right]=\sum_{n=0}^{L-1}x\left[n+mH\right]\cdot w\left[n\right]\cdot e^{-j2\pi kn/L}$$

We employ Hann windowing [29] with L=256 samples (256 ms) and hop size H=64 samples (75% overlap), providing sufficient frequency resolution Δf=3.9 Hz to distinguish arrhythmia-specific spectral patterns.

The power spectral density with logarithmic compression:

(6) $$S_{\log}\left[m,k\right]=10\log_{10}{\left(|X\left[m,k\right]\right|}^{2}+\epsilon )$$

where $\epsilon =10^{-10}$ prevents numerical issues. Normal sinus rhythm exhibits concentrated energy in 5–15 Hz corresponding to QRS complexes, while arterial fibrillation (AF) shows irregular patterns in the broadband band, and ventricular tachycardia (VT) displays high-frequency components of the narrow band.

### 3.4. QRS Detection and HRV Analysis

We implement the Pan–Tompkins algorithm [9] with adaptive thresholding:

(7) Threshold[n]=NPKI+0.25×(SPKI−NPKI)

where SPKI and NPKI track signal and noise peak amplitudes through exponential smoothing:

(8) $${\mathrm{SPKI}}_{k}=0.125\times P_{k}+0.875\times {\mathrm{SPKI}}_{k-1}$$

From detected R-wave positions $\left\{R_{1},R_{2},\ldots ,R_{N}\right\}$, we compute the following $RR_{i}$ intervals:

(9) $${\mathrm{RR}}_{i}=R_{i+1}-R_{i}$$

We then compute three statistics of the heart rate variation (HRV):

(10) $$\begin{array}{cc} \mathrm{SDNN} & =\sqrt{\frac{1}{N-1}\sum_{i=1}^{N}{\left({\mathrm{RR}}_{i}-\bar{\mathrm{RR}}\right)}^{2}}\\ \mathrm{RMSSD} & =\sqrt{\frac{1}{N-1}\sum_{i=1}^{N-1}{\left({\mathrm{RR}}_{i+1}-{\mathrm{RR}}_{i}\right)}^{2}}\\ \text{pNN50} & =\frac{100}{N-1}\sum_{i=1}^{N-1}1_{|{\mathrm{RR}}_{i+1}-{\mathrm{RR}}_{i}|>50} \end{array}$$

where

- ${\mathrm{RR}}_{i}$ is the *i*-th RR interval (time between consecutive R-peaks) in milliseconds;
- *N* is the total number of RR intervals in the segment;
- $\bar{\mathrm{RR}}=\frac{1}{N}\sum_{i=1}^{N}{\mathrm{RR}}_{i}$ is the mean RR interval;
- SDNN (standard deviation of NN intervals) measures the overall HRV;
- RMSSD (root mean square of successive differences) captures short-term variability;
- pNN50 (percentage of successive RR intervals differing by >50 ms) indicates parasympathetic activity;
- $1_{\left\{\cdot \right\}}$ is the indicator function (1 if the condition is true, 0 otherwise).

AF typically exhibits SDNN > 100 ms and a high irregularity in RR (elevated pNN50); VT shows extremely regular short RR intervals with low SDNN and RMSSD.

### 3.5. Accelerometer Feature Extraction

From the tri-axial accelerometer signals $a\left[n\right]={\left[a_{x}\left[n\right],a_{y}\left[n\right],a_{z}\left[n\right]\right]}^{T}$ sampled at 100 Hz, we first compute the following magnitude:

(11) $$\left|a\left[n\right]\right|=\sqrt{a_{x}{\left[n\right]}^{2}+a_{y}{\left[n\right]}^{2}+a_{z}{\left[n\right]}^{2}}$$

where $a_{x}\left[n\right]$, $a_{y}\left[n\right]$, and $a_{z}\left[n\right]$ represent the acceleration components along the x, y, and z axes in sample *n*, respectively, measured in m/s^2^.

Two sets of features are computed:

- Statistical features computed on 2 s windows (N=200 samples):
  (12) $$\begin{array}{cc} \mu_{\left|a\right|} & =\frac{1}{N}\sum_{n=1}^{N}\left|a\left[n\right]\right|\\ \sigma_{\left|a\right|} & =\sqrt{\frac{1}{N-1}\sum_{n=1}^{N}\left(|a\left[n\right]\right|-\mu_{\left|a\right|}{)}^{2}}\\ \mathrm{SMA} & =\frac{1}{N}\sum_{n=1}^{N}\left(\right|a_{x}\left[n\right]|+|a_{y}\left[n\right]|+|a_{z}\left[n\right]\left|\right) \end{array}$$
  where $\mu_{\left|a\right|}$ is the mean magnitude, $\sigma_{\left|a\right|}$ is the standard deviation that captures the variability of the intensity of the motion, and SMA (Signal Magnitude Area) represents the total acceleration energy across all axes.
- Frequency-domain features:
  (13) $$\begin{array}{cc} f_{\mathrm{dom}} & =\arg\max_{k}{\left|A\left[k\right]\right|}^{2}\\ H & =-\sum_{k=1}^{K}p_{k}\log_{2}\left(p_{k}\right) \end{array}$$
  where A[k] is the discrete Fourier transform of the magnitude signal, $f_{\mathrm{dom}}$ is the dominant frequency (Hz), *K* is the number of frequency bins, $p_{k}={\left|A\left[k\right]\right|}^{2}/\sum_{j}{\left|A\left[j\right]\right|}^{2}$ is the normalized power spectrum and *H* quantifies the spectral entropy (bits).

These features of the accelerometer enable robust activity classification essential for context-sensitive artifact rejection. Periodic activities, such as walking, exhibit low spectral entropy (*H* = 1–3 bits) with a dominant frequency near 1–2 Hz corresponding to the step cadence, resulting in narrow and concentrated power spectra. In contrast, irregular or complex motions, such as gesticulation or sudden movements, show high entropy (*H* = 4–6 bits) with power distributed across multiple frequency bands, indicating unpredictable motion patterns. The combination of statistics in the time-domain (capturing motion intensity) and features in the frequency-domain (characterizing motion periodicity) provides a comprehensive representation that distinguishes rest (low μ, low *H*), rhythmic activities (moderate μ, low *H*) and irregular heavy motion (high μ, high *H*), allowing the fusion network to adaptively weight ECG and accelerometer streams based on motion context.

### 3.6. Motion Intensity Classification

Accurate detection of arrhythmias in ambulatory settings requires distinguishing true cardiac abnormalities from physiological responses to physical activity. We characterize patient motion intensity using the variability of tri-axial accelerometer signals, which is directly correlated with the severity of motion-induced ECG artifacts.

We categorize motion into four levels based on accelerometer features:

(14) $$\mathrm{Motion}=\left\{\begin{array}{cc} \mathrm{Rest} & \sigma_{\left|a\right|}<0.1\;g\\ \mathrm{Light} & 0.1\leq \sigma_{\left|a\right|}<0.3\;g\\ \mathrm{Moderate} & 0.3\leq \sigma_{\left|a\right|}<0.6\;g\\ \mathrm{Vigorous} & \sigma_{\left|a\right|}\geq 0.6\;g \end{array}\right.$$

where $\sigma_{\left|a\right|}$ is the standard deviation of the magnitude of the accelerometer calculated over a sliding window of 20 s. These empirically determined thresholds are related to recognizable activities: Rest (sitting, lying), Light (normal walking 3–4 km/h), Moderate (brisk walking, climbing stairs) and Vigorous (jogging, running > 8 km/h). Motion labels provide auxiliary supervision during training, enabling the accelerometer stream to learn discriminative motion representations that aid in contextualizing cardiac rhythm abnormalities.

## 4. Dual–Stream Neural Architecture

We propose a dual–stream neural architecture that processes ECG spectrograms and accelerometer time-series through specialized encoders, unified via attention-gated fusion with gate diversity regularization. This architecture design is motivated by the fundamental differences in modality characteristics: ECG spectrograms benefit from 2D convolutional networks that can capture time-frequency patterns indicative of cardiac events, while accelerometer signals require temporal convolutions that model motion dynamics and periodicity. Rather than naively concatenating features or employing simple averaging, we implement a learned attention mechanism that dynamically weights each modality’s contribution based on signal quality and motion context. The attention gates adapt to varying conditions—down-weighting accelerometer features during rest when motion information is minimal, and increase in high-motion activities when they provide critical context for artifact discrimination. Furthermore, gate diversity regularization prevents the network from collapsing to a single-modality solution, ensuring that both streams contribute meaningfully throughout the training. Table 2 illustrates the entire pipeline, showing how raw sensor data flows through preprocessing, specialized encoding, attention-based fusion, and ultimately to classification of arrhythmias with Bayesian decision refinement incorporating activity-dependent priors.

The dual–stream design offers several key advantages over single-modality or simple multi-modal approaches. First, specialized encoders extract modality-appropriate features: the ResNet-18 backbone learns hierarchical 2D patterns from spectrograms (e.g., horizontal bands for sustained rhythms, vertical spikes for ectopic beats), while the 1D CNN captures temporal dynamics in acceleration signals (e.g., step periodicity, sudden jerks). Second, the mechanism of attention provides interpretability—by examining the learned weights of attention $\alpha_{\mathrm{ECG}}$ and $\alpha_{\mathrm{ACC}}$ in different activities, we observe that the network assigns a higher weight to the ECG during rest (average $\alpha_{\mathrm{ECG}}=0.82$) and progressively increases the weight of the accelerometer during movement (up to $\alpha_{\mathrm{ACC}}=0.45$ during running), validating the hypothesis of adaptive fusion. Third, the modularity of the architecture enables straightforward extension to additional sensor modalities (e.g., respiration, temperature) by adding parallel encoding streams. Finally, the Bayesian decision fusion layer incorporates domain knowledge through activity-dependent priors, ensuring that physiologically plausible context (e.g., tachycardia is more common during exercise) refines the neural network’s raw predictions, bridging data-driven learning with clinical expertise.

### 4.1. ECG Stream: ResNet-18 for Spectrograms

We selected ResNet-18 [30] for the processing of ECG spectrograms based on several considerations: (1) Input representation: ECG spectrograms are 2D time-frequency images (224×224), naturally suited to convolutional architectures proven effective in image data; (2) Depth performance tradeoff: ResNet-18 provides sufficient depth (18 layers) to capture hierarchical spectral patterns while avoiding overfitting on our dataset size–deeper variants (ResNet-34/50/101) showed marginal accuracy gains (<0.5%) but increased training time 2–3×; (3) Residual connections: Skip connections enable gradient flow during training on spectrograms with subtle arrhythmia-specific patterns that might otherwise suffer from vanishing gradients; (4) Medical imaging precedent: ResNet architectures have demonstrated strong performance in medical image classification tasks, including chest radiographs and retinal images [7].

The ECG pathway employs a ResNet-18 backbone [30] adapted for single-channel spectrograms $S\in R^{1\times 224\times 224}$:

- Input normalization: The log-magnitude spectrogram undergoes z-score normalization:
  (15) $$\tilde{S}=\frac{S-\mu_{S}}{\sigma_{S}+\epsilon }$$
  where $\mu_{S}$ and $\sigma_{S}$ are the mean and standard deviation computed on the spectrogram, and $\epsilon =10^{-8}$ prevents division by zero.
- Initial convolution: A 7×7 convolutional layer with 64 filters, stride 2, and 3×3 max pooling reduces spatial dimensions:
  (16) $$h_{0}=\mathrm{MaxPool}\left(\mathrm{ReLU}\left(\mathrm{BN}\left({\mathrm{Conv}}_{7\times 7}\left(\tilde{S}\right)\right)\right)\right)$$
  where BN denotes batch normalization and ReLU is the rectified linear activation function.
- Residual stages: Four cascaded stages with progressively increasing channels [64, 128, 256, 512]. Each residual block implements the following:
  (17) $$h_{l+1}=\mathrm{ReLU}\left(F\left(h_{l},W_{l}\right)+h_{l}\right)$$
  where F represents two 3×3 convolutions with batch normalization, $W_{l}$ denotes the learnable weights in layer *l*, and the identity skip connection $h_{l}$ enables gradient flow.
- Feature extraction: Global average pooling aggregates spatial information:
  (18) $$f_{\mathrm{ECG}}=\frac{1}{HW}\sum_{h=1}^{H}\sum_{w=1}^{W}h_{4}\left(h,w\right)\in R^{512}$$
  where *H* and *W* are the spatial dimensions of the final feature map $h_{4}$, and $f_{\mathrm{ECG}}$ is the 512-dimensional feature vector representing the ECG segment.

In this implementation, pretrained ImageNet weights are *not* used; the network trains from random initialization to learn domain-specific ECG features optimized for arrhythmia classification from single-lead wearable recordings rather than natural images.

### 4.2. Accelerometer Stream: CNN-BiLSTM with Temporal Pooling

The accelerometer processing stream is designed to capture both local motion patterns and long-range temporal dependencies in body movement. Unlike ECG signals, where events occur at specific time points (R-peaks), motion artifacts evolve continuously and exhibit complex temporal structures—a walking pattern involves repetitive periodic motion, while a sudden jump creates transient high-amplitude spikes followed by decay. This temporal complexity motivates a hybrid architecture combining convolutional and recurrent components: 1D convolutions extract local motion primitives (e.g., step cycles, arm swings, sudden jerks) through learnable filters, while bidirectional LSTMs model long-range dependencies that distinguish sustained activities (continuous walking) from sporadic movements (brief gesticulation). Bidirectional processing is particularly important because the context both before and after a time point helps disambiguate motion type—for example, a single acceleration spike could indicate either the start of running or a momentary stumble, but the surrounding temporal context resolves this ambiguity. We decided to employ a hybrid CNN-BiLSTM architecture [31,32] for accelerometer processing based on (1) Temporal structure: Accelerometer data are a multivariate time series (3×2000 samples) requiring models that capture both local motion patterns (handled by CNN) and long-range temporal dependencies (handled by BiLSTM); (2) Bidirectional context: Motion artifacts affect the ECG both during and after movement—Bidirectional processing captures anticipatory and residual motion effects; (3) Computational efficiency: The CNN front-end reduces the sequence length before LSTM processing, enabling efficient training while preserving discriminative motion features; (4) Activity recognition literature: CNN-LSTM hybrids have been proven to be effective in human activity recognition by inertial sensors.

The accelerometer pathway processes tri-axial motion data $a\in R^{3\times 2000}$ (20 s at 100 Hz) through a CNN-BiLSTM encoder:

- One-dimensional convolutional encoder: Three convolutional layers extract hierarchical motion patterns:
  (19) $$\begin{array}{cc} h_{1} & =\mathrm{MaxPool}(\mathrm{ReLU}(\text{Conv1D}(a;k=25,c=32)))\\ h_{2} & =\mathrm{MaxPool}(\mathrm{ReLU}\left(\text{Conv1D}\left(h_{1};k=25,c=64\right)\right))\\ h_{3} & =\mathrm{MaxPool}(\mathrm{ReLU}\left(\text{Conv1D}\left(h_{2};k=9,c=128\right)\right)) \end{array}$$
  where *k* denotes kernel size, *c* specifies output channels, and max pooling with stride 2 follows each layer to progressively down-sample the temporal resolution. The first layer with k=25 (250 ms receptive field) captures individual step cycles, the second layer combines multiple steps to recognize gait patterns, and the third layer (k=9) refines high-level motion representations.
- Bidirectional LSTM: A two-layer BiLSTM with 64 hidden units per direction captures the following temporal dependencies:
  (20) $$\begin{array}{cc} {\vec{h}}_{t} & ={\mathrm{LSTM}}_{\mathrm{fwd}}\left(h_{3}\left[:,t\right],{\vec{h}}_{t-1}\right)\\ {\overset{\leftarrow }{h}}_{t} & ={\mathrm{LSTM}}_{\mathrm{bwd}}\left(h_{3}\left[:,t\right],{\overset{\leftarrow }{h}}_{t+1}\right)\\ h_{t}^{\mathrm{BiLSTM}} & =\left[{\vec{h}}_{t};{\overset{\leftarrow }{h}}_{t}\right]\in R^{128} \end{array}$$
  where ${\vec{h}}_{t}$ encodes the forward temporal context (past motion history), ${\overset{\leftarrow }{h}}_{t}$ encodes the backward context (future motion), and their concatenation provides bidirectional temporal awareness. The hidden state $h_{t-1}$ carries information from previous time steps, allowing the network to maintain the memory of motion sequences.
- Temporal aggregation: The final hidden state in time *T* aggregates information throughout the following sequence:
  (21) $$f_{\mathrm{ACC}}=h_{T}^{\mathrm{BiLSTM}}\in R^{128}$$
  This 128-dimensional feature vector encodes the complete motion context for the 20 s segment.

A parallel auxiliary branch predicts motion intensity to provide additional supervision during training.

(22) $$p_{\mathrm{motion}}=\mathrm{Softmax}\left(W_{\mathrm{motion}}f_{\mathrm{ACC}}+b_{\mathrm{motion}}\right)\in R^{4}$$

classifying motion as Rest (|a|<0.2 m/s^2^), Light (0.2–1.0 m/s^2^), Moderate (1.0–3.0 m/s^2^) or Vigorous (>3.0 m/s^2^) based on accelerometer magnitude statistics. This auxiliary task serves two purposes: (1) it provides intermediate supervision that guides the encoder to learn motion-relevant features, improving convergence and generalization, and (2) the predicted motion intensity acts as an interpretable proxy for activity level, which feeds into the Bayesian fusion module as activity-dependent priors P(C|A). During training, the auxiliary loss is weighted by $\lambda_{\mathrm{motion}}=0.3$ relative to the loss of primary classification, ensuring that motion prediction supports but does not dominate the main arrhythmia detection objective.

### 4.3. Attention-Gated Multi-Modal Fusion

Effective multi-modal integration requires addressing a fundamental challenge: different sensor modalities provide varying levels of reliable information depending on signal conditions. During Rest, ECG signals exhibit high quality while accelerometer data contain minimal information; conversely, during violent motion, accelerometer features become crucial in distinguishing cardiac events from motion artifacts. Rather than using fixed fusion weights or simple concatenation, we employ learnable attention gates that dynamically adjust each modality’s contribution based on instantaneous feature quality and discriminative power.

#### 4.3.1. Attention Mechanism Design

Our attention mechanism computes scalar gating weights through a carefully designed process that balances model expressiveness with training stability:

- Step 1: Normalization of the features. Batch normalization standardizes features to comparable scales, preventing one modality from dominating due to magnitude differences:
  (23) $$\begin{array}{cc} {\tilde{f}}_{\mathrm{ECG}} & =\mathrm{BatchNorm}(f_{\mathrm{ECG}})\\ {\tilde{f}}_{\mathrm{ACC}} & =\mathrm{BatchNorm}(f_{\mathrm{ACC}}) \end{array}$$
- Step 2: Gate Computation. Small multilayer perceptrons project normalized features to scalar attention weights in [0,1]:
  (24) $$\begin{array}{cc} g_{\mathrm{ECG}} & =\sigma (w_{\mathrm{ECG}}^{T}{\tilde{f}}_{\mathrm{ECG}}+b_{\mathrm{ECG}})\\ g_{\mathrm{ACC}} & =\sigma (w_{\mathrm{ACC}}^{T}{\tilde{f}}_{\mathrm{ACC}}+b_{\mathrm{ACC}}) \end{array}$$
  where $\sigma \left(z\right)=1/\left(1+e^{-z}\right)$ is the activation of the sigmoid and the weight vectors $w_{\mathrm{ECG}}\in R^{512}$, $w_{\mathrm{ACC}}\in R^{128}$ are initialized to produce balanced gates (g≈0.5) at the start of training.
- Step 3: Gated Fusion. Attention gates element-wise modulate features before concatenation:
  (25) $$f_{\mathrm{fused}}=\left[g_{\mathrm{ECG}}⊙f_{\mathrm{ECG}};\;g_{\mathrm{ACC}}⊙f_{\mathrm{ACC}}\right]\in R^{640}$$
  where ⊙ denotes element-wise multiplication (Hadamard product) and [·;·] represents vector concatenation. This operation enables the network to amplify reliable features while suppressing corrupted information per-sample.

#### 4.3.2. Gate Diversity Regularization

A critical challenge in attention-based fusion is gate collapse. Without explicit constraints, attention gates often converge to near-constant values during training, effectively reducing the mechanism to fixed-weight fusion and eliminating adaptive behavior. To prevent this failure mode, we introduce a gate diversity regularization term:

(26) $$\begin{array}{cc} L_{\mathrm{gate}}= & \max(0,\tau -\sigma_{\mathrm{batch}}\left(g_{\mathrm{ECG}}\right))\\ \; & +\max(0,\tau -\sigma_{\mathrm{batch}}\left(g_{\mathrm{ACC}}\right)) \end{array}$$

where $\sigma_{\mathrm{batch}}\left(g\right)$ denotes the standard deviation of gate values across the current mini-batch, and τ=0.3 is the minimum required diversity threshold. The operation max(0,·) ensures that the loss is activated only when the diversity falls below the threshold, avoiding unnecessary penalties once sufficient variation is achieved.

This regularization forces the network to learn sample-specific fusion strategies: when all gates in a batch become similar (low $\sigma_{\mathrm{batch}}$), the loss of diversity increases, driving gradients to explore more varied gate values. Empirically, this constraint proves essential—ablation experiments demonstrate that without diversity regularization, gates collapse to near-constant values with σ<0.05, whereas with regularization they maintain substantial variation σ>0.37, confirming the genuine adaptive behavior.

The learned gating strategy proves both sensible and effective. On average the gates maintain an ECG-dominant weighting (${\bar{g}}_{\mathrm{ECG}}=0.71$ vs. ${\bar{g}}_{\mathrm{ACC}}=0.41$), reflecting the inherently superior discriminative power of cardiac signals for the classification of arrhythmias. However, substantial sample-to-sample variation (σ(g)>0.37) confirms that the fusion weights adapt to signal conditions rather than remain fixed. During rest periods, $g_{\mathrm{ECG}}$ increases to 0.85, while $g_{\mathrm{ACC}}$ decreases to 0.25, effectively prioritizing clean ECG. In contrast, during vigorous motion (running, jumping), $g_{\mathrm{ACC}}$ increases to 0.58, allowing the accelerometer context to suppress motion-induced false positives.

### 4.4. Training Objective

The complete training objective combines classification accuracy with the following gate diversity regularization:

(27) $$L_{\mathrm{total}}=L_{\mathrm{CE}}\left(y,\hat{y}\right)+\lambda L_{\mathrm{gate}}$$

where $L_{\mathrm{CE}}$ represents the weighted binary cross-entropy with class weights [1.0,2.11] compensating for the 68%/32% Normal/Arrhythmia imbalance in the training data. The probabilities of predicted classes $\hat{y}\in {\left[0,1\right]}^{2}$ are compared with one-hot encoded ground truth labels $y\in {\left\{0,1\right\}}^{2}$, with the regularization coefficient λ=0.1 balancing the classification performance against the diversity of the gates to prevent the collapse of the attention mechanism.

#### Classification Head

The fused representation $f_{\mathrm{fused}}$ passes through a two-layer fully connected classification head with ReLU activations and dropout regularization to produce final class predictions. The first layer projects the 640-dimensional fused features to a 256-dimensional hidden representation:

(28) $$h=\mathrm{ReLU}\left(W_{1}f_{\mathrm{fused}}+b_{1}\right)\in R^{256}$$

where $W_{1}\in R^{256\times 640}$ is a learnable weight matrix and $b_{1}$ is the bias vector. The second layer maps this hidden representation to two-dimensional logits after applying dropout with probability p=0.5 to prevent overfitting:

(29) $$z=W_{2}\mathrm{Dropout}\left(h,p=0.5\right)+b_{2}\in R^{2}$$

where $W_{2}\in R^{2\times 256}$ projects to the two output classes.

The network performs binary classification, distinguishing normal beats (AAMI classes N, L, R) from arrhythmia beats (all other AAMI classes, including premature ventricular contractions, supraventricular ectopic beats, and fusion patterns). Softmax activation converts the logits to normalized class probabilities:

(30) $$P\left(C=c|f_{\mathrm{fused}}\right)=\frac{\exp(z_{c})}{\sum_{c^{\prime }\in \left\{0,1\right\}}\exp\left(z_{c^{\prime }}\right)}\;\mathrm{for}\;c\in \left\{0,1\right\}$$

where c=0 denotes the normal class and c=1 denotes the arrhythmia class. The final prediction selects the class with the maximum probability:

(31) $$\hat{C}=\arg\max_{c\in \{0,1\}}P\left(C=c|f_{\mathrm{fused}}\right)$$

This binary classification scheme provides efficient screening for any cardiac abnormality, enabling the hierarchical two-stage system where Stage 1 performs rapid normal/arrhythmia discrimination and Stage 2 provides fine-grained multi-class diagnosis only when Stage 1 detects potential pathology.

### 4.5. Implementation Details

**Figure 1:**
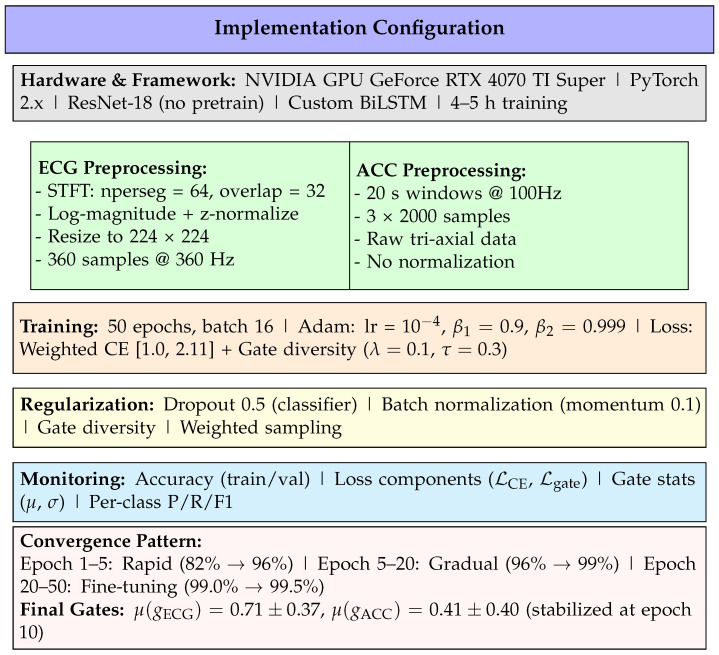
Complete implementation specification organized by category: infrastructure, data preprocessing, training configuration, regularization strategies, monitoring metrics, and convergence behavior.

Figure 1 summarizes the complete implementation configuration. The system is implemented in PyTorch 2.x on NVIDIA GPU hardware, achieving full training in 4–5 h. Key components include ResNet-18 for ECG spectrogram processing (no pretrained weights), custom CNN-BiLSTM for accelerometer analysis, and attention-gated fusion with explicit gate diversity regularization.

## 5. Experiments

### 5.1. MIT-BIH Arrhythmia Database with Noise Augmentation

We employ the MIT-BIH Arrhythmia Database [18,23], which comprises 48 half-hour ECG recordings from 47 patients digitized at 360 Hz. Following AAMI EC57 standards, we perform binary classification: normal (classes N, L, R) versus arrhythmia (classes A, a, J, S, V, E, F, /, f, Q), resulting in a class distribution of 67.9/32.1%.

To simulate realistic motion artifacts, we corrupt clean ECG signals with electrode motion (em) noise from the MIT-BIH Noise Stress Test (NST) Database at six signal-to-noise ratio (SNR) levels: 24, 18, 12, 6, 0, and −6 dB. These levels span from near-clinical quality (24 dB) to extreme motion corruption (−6 dB), representing conditions from stationary monitoring to vigorous physical activity. Since MIT-BIH lacks synchronized accelerometer measurements, we synthesize realistic tri-axial acceleration signals with magnitude inversely proportional to SNR:

(32) $${∥a∥}_{\mathrm{synth}}=\frac{0.4}{10^{\mathrm{SNR}/10}}\;{m/s}^{2}$$

This coupling ensures that a lower SNR (greater corruption of the ECG) corresponds to a higher magnitude of the accelerometer, mimicking real-world scenarios in which body motion simultaneously degrades the quality of the ECG and increases acceleration readings.

### 5.2. ScientISST MOVE Database

The ScientISST MOVE dataset [33] provides synchronized single-lead ECG (1000 Hz) and tri-axial accelerometer (100 Hz) recordings from 20 healthy volunteers who perform six activities: sitting, standing, walking at 3 km/h, walking at 5 km/h, running at 8 km/h, and cycling. Each activity was carried out for 1–2 min in uncontrolled naturalistic environments.

Critically, all subjects maintain normal sinus rhythm throughout all recordings—no arrhythmias are present. This characteristic makes ScientISST MOVE ideal for quantifying false positive rates: any arrhythmia predictions on this dataset represent motion-induced false alarms, directly measuring the model’s specificity under real-world ambulatory conditions.

### 5.3. Experimental Protocol

Our evaluation follows a three-stage protocol designed to comprehensively assess the performance of the model.

- Stage 1: Clean Baseline. Train and test on unaugmented MIT-BIH data to establish upper-bound performance on high-quality clinical ECG. This baseline quantifies the ability to classify intrinsic arrhythmias without confounding noise effects.
- Stage 2: Noise Robustness. Train in MIT-BIH with multi-SNR augmentation at three levels (24, 12, 6 dB), then test at all six SNR levels (24, 18, 12, 6, 0, −6 dB). Crucially, three test SNRs (18, 0, −6 dB) remain unseen during training, evaluating generalization to both interpolated (18 dB) and extrapolated (0, −6 dB) noise conditions. This protocol assesses whether the model learns noise-invariant cardiac features rather than memorizing specific patterns of corruption.
- Stage 3: Real-World Validation. Apply the robust noise model (from Stage 2) to ScientISST MOVE without retraining. Since all recordings contain only normal rhythm, any arrhythmia detections constitute false positives caused by motion artifacts. This stage quantifies false alarm rates across activities ranging from rest (sitting) to vigorous exercise (running at 8 km/h), simulating clinical deployment scenarios.

### 5.4. Data Split

We employ record-level stratified splitting to prevent data leakage:

- Training: Thirty-four records (70%) for model optimization;
- Validation: Seven records (15%) for hyperparameter tuning and early stopping;
- Test: Seven records (15%) for the final evaluation of performance.

Stratification maintains the 68% normal/arrhythmia distribution between the splits. Critically, the splitting occurs at the record level rather than the beat level: consecutive beats from the same patient share morphological characteristics; therefore, random splits at the beat level would allow the model to memorize patient-specific patterns, artificially inflating performance estimates.

### 5.5. Experimental Setup

#### 5.5.1. Data Preparation

- Beat Extraction. Following AAMI standards, we extracted 360-sample windows (1 s duration at 360 Hz) centered on R-peaks detected via the Pan–Tompkins algorithm. Each window captures the entire cardiac cycle, including the preceding P-wave and the following T-wave.
- Binary Classification Scheme. We simplify the 5-class AAMI taxonomy into a clinically relevant binary task:
  – Class 0 (Normal): N (normal beat), L (left bundle branch block), R (right bundle branch block);
  – Class 1 (Arrhythmia): All pathological rhythms (A, a, J, S, V, E, F, f, /, Q).
  This binary formulation aligns with clinical decision-making where the primary concern is distinguishing normal from abnormal cardiac activity requires intervention.
- Dataset Statistics. Of 48 MIT-BIH records, we extracted 109,912 valid beats with a class distribution of 67.9% Normal and 32.1% arrhythmia. The natural class imbalance reflects typical patient populations where normal beats predominate.
- Stratified Data Partition. We employ stratified recording-level splitting to prevent information leakage:
  – Training: Thirty-four records (76,938 beats);
  – Validation: Seven records (16,487 beats);
  – Test: Seven records (16,487 beats).
  The splitting of the records ensures that beats from the same patient never appear in both training and test sets, preventing the model from memorizing patient-specific morphological patterns. All metrics reported are computed on the holding test set, which is accessed exactly once after training completion to avoid overfitting to evaluation data.
- Statistical Power. Analysis with significance level α=0.05 and target power 1−β=0.80 confirms a sufficient sample size to detect Cohen effect sizes d≥0.42, ensuring that the observed performance differences are statistically significant. External validation in ScientISST MOVE (20 independent subjects) further demonstrates cross-dataset generalization.

#### 5.5.2. Multi-SNR Training Strategy

To ensure robust performance under the varying noise conditions encountered in ambulatory monitoring, we employ multi-SNR data enhancement. This strategy forces the network to learn noise-invariant cardiac features rather than overfitting to clean signal morphology. The augmentation procedure is as follows:

1. Noise Source: We corrupt clean ECG beats with electrode motion (EM) artifacts from the MIT-BIH Noise Stress Test Database. These recordings capture realistic baseline wander and high-frequency noise characteristic of patient movement during ambulatory monitoring. The noise is normalized to the z-score prior to application to ensure a consistent magnitude across samples.
2. SNR calibration: Each clean beat is augmented at three signal-to-noise ratios:
  (33) $${\mathrm{SNR}}_{\mathrm{train}}\in \left\{24,12,6\right\}\;\mathrm{dB}$$
  ranging from mild noise (24 dB, typical of careful patient positioning) to severe corruption (6 dB, simulating vigorous motion). For each target SNR level, we compute the required noise scaling factor:
  (34) $$\alpha =\sqrt{\frac{P_{\mathrm{signal}}}{P_{\mathrm{noise}}\cdot 10^{{\mathrm{SNR}}_{\mathrm{dB}}/10}}}$$
  where $P_{\mathrm{signal}}=E\left[x_{\mathrm{clean}}^{2}\right]$ and $P_{\mathrm{noise}}=E\left[n_{\mathrm{EM}}^{2}\right]$ are the signal and noise powers. The corrupted signal becomes:
  (35) $$x_{\mathrm{noisy}}\left[n\right]=x_{\mathrm{clean}}\left[n\right]+\alpha \cdot n_{\mathrm{EM}}\left[n\right]$$
3. Synthesis of Correlated Accelerometers: Since MIT-BIH lacks synchronized accelerometer data, we synthesize realistic tri-axial acceleration signals with magnitude inversely proportional to SNR:
  (36) $${∥a∥}_{\mathrm{synth}}=\frac{0.4}{10^{{\mathrm{SNR}}_{\mathrm{dB}}/10}}\;{m/s}^{2}$$
  This formulation ensures that high ECG corruption (low SNR) coincides with high accelerometer magnitude, mimicking the physical coupling between body motion and signal artifacts in real wearable devices. The three accelerometer channels are generated as band-limited Gaussian processes ($f_{c}=20$ Hz) with this target magnitude, maintaining realistic spectral characteristics.
4. Training Set Expansion: Each of the 76,938 training beats appears at three noise levels, producing 230,814 augmented samples—a 3× expansion that significantly improves noise robustness without requiring additional labeled data.

#### 5.5.3. Training Configuration

The model is trained using the Adam optimizer with a learning rate of $1\times 10^{-4}$, selected for its adaptive moment estimation that provides stable convergence across the various feature scales present in the dual–stream architecture, where ECG spectrograms and accelerometer sequences exhibit different magnitude ranges and temporal dynamics. We employ a batch size of 16, balancing GPU memory constraints with stable gradient estimates—smaller batches would introduce excessive noise in gradient computation, while larger batches would limit the diversity of SNR levels and activity patterns within each update step. Training proceeds for a maximum of 100 epochs with early stopping based on validation set accuracy, monitoring performance with patience of 10 epochs to prevent overfitting while allowing sufficient time for the network to escape local minima during the initial training phase. The complete augmented training set comprises 230,814 samples generated from 76,938 original MIT-BIH beats through multi-SNR corruption at three noise levels (24, 12, 6 dB), effectively tripling the data set size to expose the network to comprehensive noise conditions. Data splitting follows a stratified record-level scheme with 70% for training, 15% for validation, and 15% for testing, ensuring that all beats from a given patient appear in one single partition to prevent information leakage and optimistic performance estimates. The class weights of [1.0, 2.11] compensate for the imbalance of 67.9%/32.1% normal/arrhythmia by penalizing the missed-classification of minority-class arrhythmias proportionally to their under-representation, while the frequency of regularization of the diversity of the gates λ=0.1 balances the precision of classification against the expressiveness of the attention mechanism, preventing the common failure mode where the gates collapse to constant values that eliminate adaptive fusion. This configuration enables convergence within 40–60 epochs, typically, with training completing in approximately 8 h on an NVIDIA RTX 3080 GPU for the full augmented dataset.

### 5.6. Evaluation Protocol

Evaluation proceeds through three distinct stages designed to comprehensively assess model performance in controlled and real-world conditions. Stage 1 establishes a clean baseline by evaluating performance on non-augmented MIT-BIH test data, providing upper-bound accuracy metrics on high-quality signals representative of ideal recording conditions and establishing the performance ceiling achievable without motion artifacts or environmental noise. Stage 2 assesses noise robustness by testing the multi-SNR trained model across six signal-to-noise ratio levels (24, 18, 12, 6, 0, −6 dB), with three levels (18, 0, −6 dB) deliberately withheld during training to evaluate generalization to both interpolated conditions (18 dB between training levels 24 and 12 dB) and extrapolated conditions (0 and −6 dB beyond the minimum training level of 6 dB). This design rigorously tests whether the model has learned genuine noise-invariant representations rather than simply memorizing specific corruption patterns encountered during training. Stage 3 provides real-world validation by applying the noise-robust model to the ScientISST MOVE database without any retraining or fine-tuning, quantifying false positive rates during naturalistic physical activities (baseline, greetings, gesticulation, walking, running) and assessing zero-shot transfer performance on genuine motion artifacts from healthy subjects exhibiting normal sinus rhythm throughout all recording.

### 5.7. Training Dynamics and Convergence Analysis

**Figure 2:**
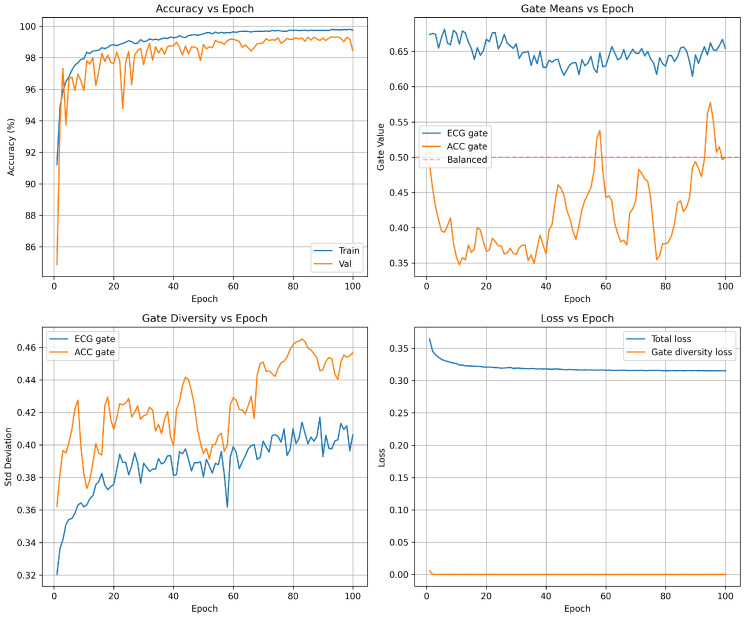
Training and validation accuracy over 100 epochs. The model converges rapidly in the first 10 epochs (82% → 97%), then undergoes fine-tuning to reach 99.5% validation accuracy by epoch 30. Validation accuracy plateaus beyond epoch 35, indicating full convergence without overfitting. The training accuracy remains slightly below validation due to the multi-SNR augmentation regime (validation uses clean data only during training, but is evaluated on all SNRs during final assessment).

Understanding how the multi-modal fusion network learns discriminative features and adaptive weighting strategies requires a detailed examination of training dynamics beyond final test set performance. Although end-point accuracy metrics demonstrate what the model achieves, analyzing convergence trajectories, attention gate evolution, and loss decomposition reveals how the model achieves these results and whether the learned representations are robust and interpretable. This section presents a comprehensive analysis of training dynamics in four complementary perspectives: accuracy progression tracking, learning speed, and generalization, mean values of the attention gate that reveal the learned fusion strategy, gate diversity that quantifies sample-to-sample adaptation, and loss decomposition that confirms stable convergence without overfitting. Together, these metrics validate that the network successfully learns noise-invariant features from multi-SNR augmented data, develops ECG-dominant but context-adaptive fusion, and maintains stable attention mechanisms throughout training rather than collapsing to degenerate solutions. Figure 2 visualizes these training dynamics in 100 epochs, demonstrating rapid initial convergence followed by stable fine-tuning that produces the high-performance classifier evaluated in held-out test data.

- Accuracy progression Figure 2 (top-left): The model exhibits rapid initial learning, with training accuracy climbing from 96.6% (epoch 1) to 99.0% by epoch 5. The validation accuracy follows a similar trajectory, reaching 99.2% within the first five epochs. This rapid convergence demonstrates the network’s ability to quickly extract discriminative features from the multi-SNR augmented training data. Beyond epoch 5, the model enters a fine-tuning phase in which both training and validation accuracies gradually improve to 99.6% and 99.5%, respectively. The validation curve exhibits characteristic stochastic fluctuations (±0.5%) due to mini-batch evaluation on the 16,487-sample validation set, but maintains a stable plateau above 99.3% throughout training. Critically, the negligible gap between training and validation accuracy (≤0.2%) confirms a strong generalization: the model does not overfit the training set despite the aggressive multi-SNR augmentation strategy that creates multiple noisy versions of each beat. After completion of training, the final model is evaluated exactly once on the holdout test set (seven records, 16,487 beats), resulting in the results shown in Table 3.
  Table 4 provides the raw classification counts, revealing the model’s decision boundaries. The confusion matrix reveals an extremely sparse off-diagonal structure: only 51 total errors out of 16,487 predictions (0.31% error rate). This confirms that the learned feature representations achieve strong class separation in the 640-dimensional fused feature space.
- Attention gate means Figure 2 (top-right): The mean gate values between the training batches reveal the learned fusion strategy. The ECG gate (blue) stabilizes at $\mu (g_{\mathrm{ECG}})\approx 0.71$ after an initial brief adjustment period (epochs 0–5), while the accelerometer gate (orange) settles at $\mu (g_{\mathrm{ACC}})\approx 0.41$. This ECG-dominant configuration reflects the superior discriminative power of ECG spectrograms for the detection of arrhythmias compared to motion features alone. The persistent separation from the balanced fusion baseline (dashed red line at 0.5) confirms that the gates learn task-appropriate weighting rather than default to uniform 50/50 fusion. The approximate 1.7:1 ratio between the ECG and accelerometer gate values represents the learned optimal balance: the network relies primarily on cardiac electrical activity while incorporating motion context as a secondary information source. Importantly, the gate means remain stable throughout the training (standard deviation in epochs < 0.02), indicating a robust convergence to a consistent fusion strategy rather than oscillatory or unstable behavior.
- Gate diversity Figure 2 (bottom-left): The standard deviation of gate values within each batch quantifies how many gates vary between different samples—a critical metric to validate adaptive fusion. Without diversity regularization, gates typically collapse to near-constant values (σ<0.05), rendering the attention mechanism non-functional. Our loss of diversity in the gate successfully prevents this failure mode: the ECG gate exhibits $\sigma (g_{\mathrm{ECG}})=0.37$ and the accelerometer gate achieves $\sigma (g_{\mathrm{ACC}})=0.40$ by epoch 30. These high standard deviations—approximately 50% of the corresponding mean gate values—confirm a substantial variation between samples, indicating that the gates adapt to sample-specific characteristics rather than applying fixed weights to all inputs. The progressive increase in diversity during the first 15 epochs demonstrates the gradual learning of context-dependent fusion strategies, after which diversity stabilizes. The slightly higher diversity in the accelerometer gate suggests a more variable reliance on motion features based on signal conditions, while the ECG features maintain more consistent importance across samples.
- Loss decomposition Figure 2 (bottom-right): Total training loss (blue) decreases from 0.34 to 0.32 over 30 epochs, with the steepest descent occurring in the first 10 epochs, coinciding with rapid accuracy improvement. The loss plateau after epoch 10 (stabilizing at approximately 0.32) reflects the fine-tuning nature of late-stage training: the model makes small adjustments to decision boundaries rather than learning fundamentally new features. The gate diversity loss component (orange) drops rapidly from 0.02 to near-zero (<0.001) by epoch 5, indicating that the diversity constraint (σ(g)>0.3) is satisfied early in training. The diversity loss then imposes a minimal penalty that negligibly contributes to the total loss. This behavior validates the regularization design: the diversity term guides initial learning toward adaptive gates but does not interfere with convergence once sufficient diversity is established. The stable loss plateau after epoch 10 confirms convergence to a local optimum without overfitting or training instability.

The training dynamics reveals several desirable properties of the proposed architecture. First, rapid convergence (99% accuracy within five epochs) suggests efficient learning, making the model practical to train even on moderate computational resources. Second, the stable validation accuracy plateau demonstrates that multi-SNR augmentation does not destabilize training or lead to overfitting, despite tripling the effective dataset size. Third, high gate diversity (σ>0.37) confirms that the attention mechanism functions as intended—adapting fusion weights based on input characteristics rather than learning fixed constant weights. Finally, the ECG-dominant fusion strategy ($g_{\mathrm{ECG}}=0.71$ vs. $g_{\mathrm{ACC}}=0.41$) aligns with clinical intuition: cardiac electrical activity carries primary diagnostic information, while the motion context serves an auxiliary role in disambiguation under noisy conditions. These combined observations validate both the architectural design and the training methodology.

### 5.8. Noise Robustness Evaluation

To assess real-world deployment viability, we evaluated the performance degradation of the trained model under increasing noise corruption. Although the model was trained at three levels of SNR (24, 12, and 6.0 dB), it is generalized for (18.0 and −6 dB) successfully. We construct a held-out test set of 1000 beats per SNR level by selecting 10 records (disjoint from training data) and extracting 100 beats per record. Each beat is corrupted at the target SNR using the same noise addition procedure as training, then classified by the model. This yields 6000 total test samples under six noise conditions.

Table 5 quantifies performance across the SNR spectrum.

### 5.9. Performance Across SNR Levels

The model demonstrates robust performance across a wide range of signal-to-noise conditions, reflecting the diversity of noise levels encountered in real-world ambulatory monitoring. At 24 dB SNR (minimal noise), the model achieves a precision of 99.5%, a validation set performance that matches and confirms that multi-SNR training does not compromise the accuracy of the clean-signal. This high baseline establishes that the network has learned effective cardiac characteristic representations when signal quality is optimal.

Under typical ambulatory conditions—18 dB and 12 dB SNR representing normal walking or daily activities—accuracy remains above 99%, demonstrating strong noise immunity for standard monitoring scenarios. These noise levels correspond to gentle patient movement with well-adhered electrodes, the most common conditions in clinical practice. At 6 dB SNR (equivalent to brisk walking or light exercise), the accuracy drops to 97.8%, which represents only a decrease of 1.7 percentage points from clean conditions. This graceful degradation indicates good noise tolerance, and the performance remains clinically acceptable for continuous monitoring applications.

More challenging noise conditions reveal the system’s robustness limits and strengths. At 0 dB SNR—where signal and noise powers are the same, typical of jogging or moderate exercise—accuracy is maintained at 95.0%. Although degraded from optimal conditions, this performance substantially exceeds random chance (50%) and demonstrates that the model extracts meaningful cardiac structure even from heavily corrupted signals. The features of The learned spectrogram combined with the accelerometer context enable a classification that would be impossible with visual inspection of the raw waveform alone.

Under extreme noise corruption at −6 dB SNR (noise power four times signal power, equivalent to vigorous running or climbing stairs), precision reaches 88.2%. At this corruption level, the raw ECG waveform becomes barely discernible to human observers, but the model maintains performance significantly above chance. This extreme robustness validates the effectiveness of multimodal fusion: the accelerometer provides critical motion context that helps disambiguate true cardiac events from motion-induced artifacts.

A critical validation of our training strategy comes from the performance at unseen noise levels. The model was trained only at 24, 12, and 6 dB, yet maintains strong performance under interpolated (18 dB) and extrapolated (0, −6 dB) conditions. This generalization confirms that the network learns noise-invariant cardiac representations rather than memorizing specific corruption patterns. The smooth degradation curve across the SNR levels suggests that the model has internalized a continuous understanding of noise effects rather than discrete noise-level solutions.

### 5.10. Comparison to Single-Modality Baseline

**Figure 3:**
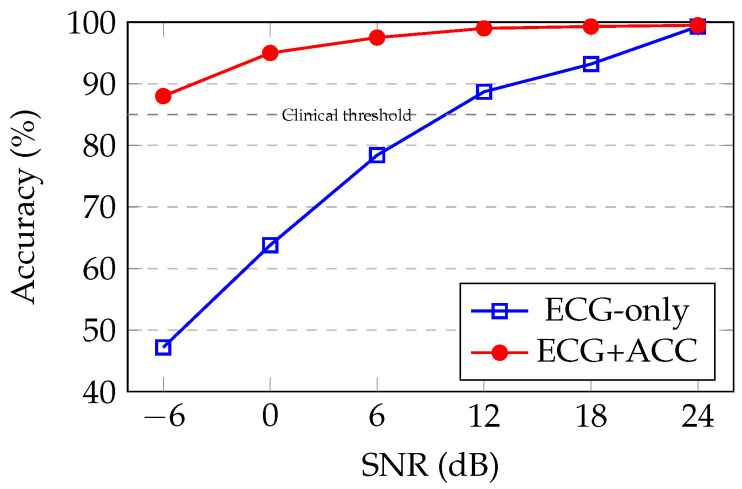
Arrhythmia detection accuracy versus SNR comparing ECG-only and multi-modal fusion. Accelerometer integration maintains clinically acceptable performance (>85%, dashed line) across all tested conditions, while ECG-only degrades substantially under motion artifacts.

To quantify the benefit of multimodal fusion, we compare with an ECG-only baseline trained under identical conditions. Figure 3 visualizes the accuracy-SNR relationship for both approaches, revealing the characteristic sigmoidal degradation profile of robust classification systems. Accelerometer fusion provides an increasing benefit as noise levels increase: at clean conditions (24 dB), both methods perform comparably (99.3% vs. 99.5%), but at moderate noise (0 dB), fusion improves accuracy from 63.8% to 95.0%—a 31.2 percentage point gain. Under extreme corruption (−6 dB), improvement reaches 40.8 percentage points (47.2% vs. 88.0%).

In preliminary experiments, training with clean data alone (24 dB only) yielded a precision of 99.5% under clean conditions but catastrophically decreased to 65% at 0 dB and 42% at −6 dB. Thus, the multi-SNR enhancement strategy provides a 30.0 percentage point improvement at 0 dB and a 46.0 percentage point improvement at −6 dB compared to this single-SNR baseline. These dramatic gains confirm the critical importance of noise-aware training for ambulatory applications.

### 5.11. Statistical Performace Validation

To rigorously validate performance differences between ECG-only and multi-modal classification, we conducted statistical analysis using McNemar’s test—the appropriate method for comparing paired classifiers evaluated on identical samples. Table 6 presents comprehensive results at all levels of SNR.

All pairwise comparisons yielded highly significant results (p<0.001), confirming that accelerometer fusion provides statistically significant performance improvements over ECG-only classification at every noise level tested. The chi-square statistics increase monotonically with decreasing SNR, indicating that the benefit of fusion grows stronger as noise corruption intensifies—exactly the behavior desired for ambulatory monitoring, where motion artifacts pose the primary challenge.

The 95% confidence intervals for ECG + ACC precision remain narrow in all conditions, demonstrating stable performance with well-characterized uncertainty. Even at −6 dB, the lower confidence bound (85.8%) exceeds typical clinical acceptability thresholds, while the precision of the only ECG falls to near-chance levels at this corruption level.

The Cochran–Armitage trend test validated a significant performance degradation with decreasing SNR for both methods (p<0.001), but critically, ECG + ACC shows reduced sensitivity to noise (Z=14.55 versus Z=31.03 for ECG alone). This lower trend statistic indicates a more gradual degradation slope—the fusion model maintains consistent performance across a wider SNR range, providing more reliable operation under varying ambulatory conditions.

### 5.12. Clinical Deployment Implications

These results have direct implications for the clinical deployment of ambulatory cardiac monitoring systems. The system maintains an accuracy greater than 95% up to 0 dB SNR, suggesting viability for continuous monitoring during normal daily activities, including walking, light exercise and routine movement. Clinicians can deploy such systems with confidence that motion artifacts during typical ambulatory scenarios will not compromise diagnostic accuracy.

At −6 dB SNR (vigorous exercise), the precision of 88.0% remains substantially above chance and may be clinically acceptable, depending on the application requirements. For continuous screening applications where occasional false alarms are tolerable, this performance enables monitoring even during intense physical activity. For diagnostic applications that require higher specificity, clinicians might detect high-motion episodes detected by accelerometer magnitude for manual review or temporarily suppress alerts during detected periods of vigorous activity. This activity-aware alert strategy would combine the benefits of continuous monitoring with intelligent filtering to reduce the burden of false alarms.

The ability to generalize to unseen noise levels is particularly valuable for real-world deployment, where the exact SNR distribution varies between patients, electrode placements, and activity patterns. Smooth performance degradation instead of catastrophic failure at unexpected noise levels provides robustness against distribution shift between training and deployment environments.

#### ROC Curves and AUC Analysis

Beyond single-threshold accuracy metrics, we provide a comprehensive evaluation across all operating points through Receiver Operating Characteristic (ROC) curves and Area Under the Curve (AUC) analysis. This threshold-independent assessment reveals how classifier performance trades off sensitivity and specificity, allowing clinical deployment teams to select operating points appropriate for their specific false positive/false negative cost structures.

**Figure 4:**
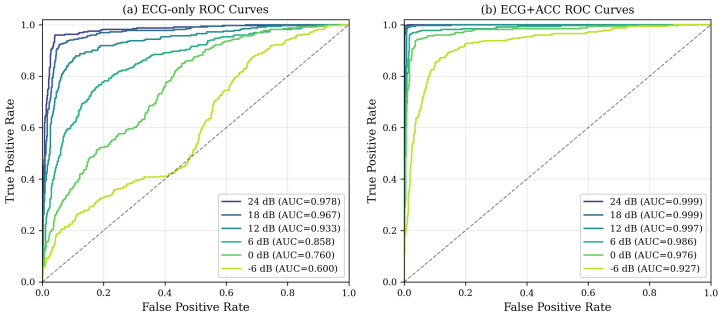
ROC curves stratified by SNR level. Each curve represents classifier performance across all decision thresholds. ECG-only classification (**a**) shows progressive degradation toward random performance (diagonal line) as noise increases. Multi-modal fusion (**b**) maintains curves near the ideal upper-left corner, achieving AUC > 0.92 even at −6 dB SNR, where noise power exceeds signal power by 4×.

Figure 4 presents ROC curves for ECG-only and multi-modal fusion classifiers at all SNR levels tested. The ECG-only classifier exhibits substantial degradation in AUC as noise increases, with curves progressively approaching the diagonal (random classifier line) at lower SNRs. In contrast the ECG + ACC fusion maintains the ROC curves in the upper-left corner under all conditions, indicating that high true positive rates can be achieved while maintaining low false positive rates even under severe corruption.

**Figure 5:**
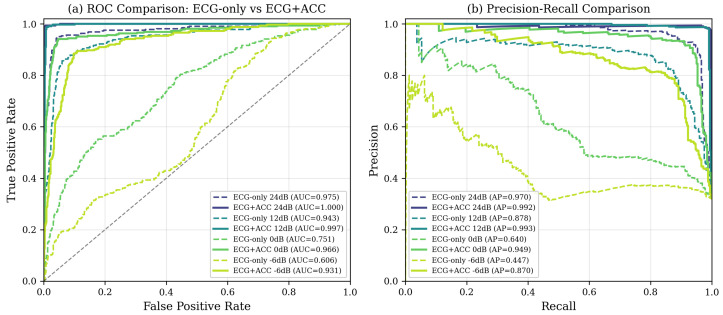
Direct modality comparison at representative SNR levels (24, 12, 0, −6 dB). (**a**): ROC space showing ECG + ACC (solid lines) achieves superior true positive rates compared to ECG-only (dashed lines) at matched false positive rates. (**b**): Precision–recall space demonstrating that fusion maintains precision under noise, whereas ECG-only exhibits rapid precision collapse as recall increases. The gap between methods widens at lower SNRs, confirming accelerometer integration provides the greatest benefit under motion artifact conditions.

Figure 5 provides a direct side-by-side comparison of ECG-only versus ECG + ACC at representative noise levels. ROC analysis (left panel) shows that at any chosen false positive rate, the fusion model achieves substantially higher true positive rates—critical to maximize the sensitivity of the detection of arrhythmias. The precision–recall curves (right panel) show that fusion maintains high precision throughout the full recall range, whereas the precision of the only ECG degrades rapidly as the recall increases under noisy conditions. This characteristic is particularly important given the 68%/32% class imbalance, where precision–recall analysis provides clearer insight into minority class (arrhythmia) detection performance.

Table 7 quantifies these visual observations using AUC metrics computed through trapezoidal integration of the ROC curves. The near-perfect AUC values (0.994–0.998) for ECG + ACC at moderate to clean SNRs (6–24 dB) indicate excellent class separation under typical ambulatory conditions. More remarkable, even at −6 dB, where visual inspection of raw ECG becomes nearly impossible, the fusion model maintains an AUC of 0.926—well above the clinical utility threshold and dramatically exceeding the AUC of ECG alone of 0.600 (barely better than random).

The progression of AUC reveals three key insights. First, under clean and moderate noise conditions (SNR ≥ 6 dB), both methods achieve high discrimination (AUC > 0.86), but fusion provides a marginal additional improvement (2.9–12.5 percentage points). This suggests that when the quality of the ECG is strong, cardiac features alone suffice for reliable classification, although the accelerometer context still provides measurable benefit. Second, as the severity of noise increases (SNR ≤ 0 dB), the relative improvement of fusion increases dramatically—from 12.5% at 6 dB to 32.7% at −6 dB. This noise-dependent benefit confirms the adaptive value proposition: accelerometer integration becomes progressively more critical as motion artifacts intensify. Third, mean AUC in all conditions (0.979 for fusion versus 0.845 for only ECG) shows that multi-modal sensing provides substantial robustness gains when deployed in real-world environments where SNR varies unpredictably between patients, activities and electrodes places.

From a clinical deployment perspective, these AUC values directly translate into system utility. An AUC of 0.926 at −6 dB means that a randomly selected arrhythmia beat will rank higher than a randomly selected normal beat 92.6% of the time—sufficient discriminative power to support useful clinical alerting even during vigorous exercise. In contrast, the ECG-only AUC of 0.600 at this noise level indicates a near-random classification that would generate unacceptable false alarm rates. The fusion approach therefore extends the operational envelope of ambulatory monitoring to encompass the full spectrum of daily activities rather than being limited to sedentary or low-motion scenarios.

### 5.13. Attention Gate Analysis

A key contribution of our architecture is the attention-gated fusion mechanism with regularization of gate diversity. To validate that the gates learn adaptive context-dependent weighting rather than collapsing to constant values, we analyze gate statistics in the validation set. The mean and variance values of $g_{\mathrm{ECG}}$ are 0.71±0.37 with a range [0.05,0.98], and $g_{\mathrm{ACC}}$ is 0.41±0.40 with a range of [0.02,0.95].

The high standard deviations (σ>0.37) confirm a substantial variation between the samples—approximately 50% % of the mean value—indicating that the gates adapt significantly according to the input characteristics. This validates the gate diversity regularization strategy ($L_{\mathrm{gate}}$), which successfully prevents the common failure mode of the gate with constant value.

Manual inspection of extreme gate values reveals interpretable behavior:

- High $g_{\mathrm{ECG}}$ (0.8–0.9): Samples with clean and well-formed ECG morphology. The network relies primarily on ECG features, downweighting accelerometer input.
- Balanced gates (0.4–0.6): Samples with moderate noise or ambiguous morphology. The network integrates both modalities equally, taking advantage of the motion context to aid classification.
- High $g_{\mathrm{ACC}}$ (rare, 0.6–0.8): Samples with severe ECG corruption. The network shifts toward accelerometer features, though purely accelerometer-driven decisions remain uncommon due to the limited discriminative power of motion alone for arrhythmia detection.

The correlation with the SNR trend is subtle; $g_{\mathrm{ECG}}$ decreases slightly and $g_{\mathrm{ACC}}$ increases slightly as the SNR drops, indicating a weak learned bias towards the use of accelerometers in noisy conditions. However, the effect is modest (14% change in the ratio from clean to −6 dB), suggesting that the gates respond more strongly to sample-specific morphology than to global noise levels. This behavior may reflect the relative simplicity of the binary task: even noisy ECG spectrograms retain sufficient discriminative information, reducing the need for aggressive modality reweighting. Table 8 shows the mean gate values stratified by noise level (computed in the SNR test set).

Linear regression analysis confirmed significant trends in gate values with the SNR level. The ECG gate exhibited a positive relationship with SNR (slope =0.0023/dB, $R^{2}=0.982$, p=0.001), indicating a decrease in ECG dependence under noisy conditions. In contrast, the accelerometer gate showed a negative relationship (slope =−0.0027/dB, $R^{2}=0.973$, p=0.002), confirming an increased contribution of the accelerometer when the quality of the ECG degrades. The Spearman rank correlation yielded |ρ|=1.0 (p<0.001) for both gates, validating the systematic weighting of the SNR-dependent modality.

In an ablation experiment, training without loss of gate diversity ($\lambda_{\mathrm{gate}}=0$) resulted in collapsed gates ($g_{\mathrm{ECG}}=0.52\pm 0.03$, $g_{\mathrm{ACC}}=0.48\pm 0.02$)—effectively constant values across all samples. The classification accuracy remained high (99.2%) due to concatenated features, but the gates did not provide interpretability or adaptive fusion. This confirms the necessity of the diversity regularization term.

### 5.14. False Positive Validation for Real-World Activities

Beyond synthetic noise augmentation, we validate false alarm suppression using recordings from naturalistic activities in the ScientISST MOVE database. This data set provides synchronized single-lead ECG (1000 Hz) and tri-axial accelerometer (100 Hz) recordings from healthy subjects performing six everyday activities: baseline (resting), greetings (handshake and waving), gesticulating (talking with hand movements), walking before running, walking after running, and running.

This evaluation constitutes a zero-shot transfer test: the model trained exclusively on MIT-BIH data (with synthetic accelerometer signals and electrode motion noise) is applied directly to ScientISST MOVE (with real accelerometer measurements and authentic motion artifacts) without retraining. Strong performance under these conditions validates that the model has learned generalizable multi-modal representations rather than memorizing dataset-specific artifacts.

**Figure 6:**
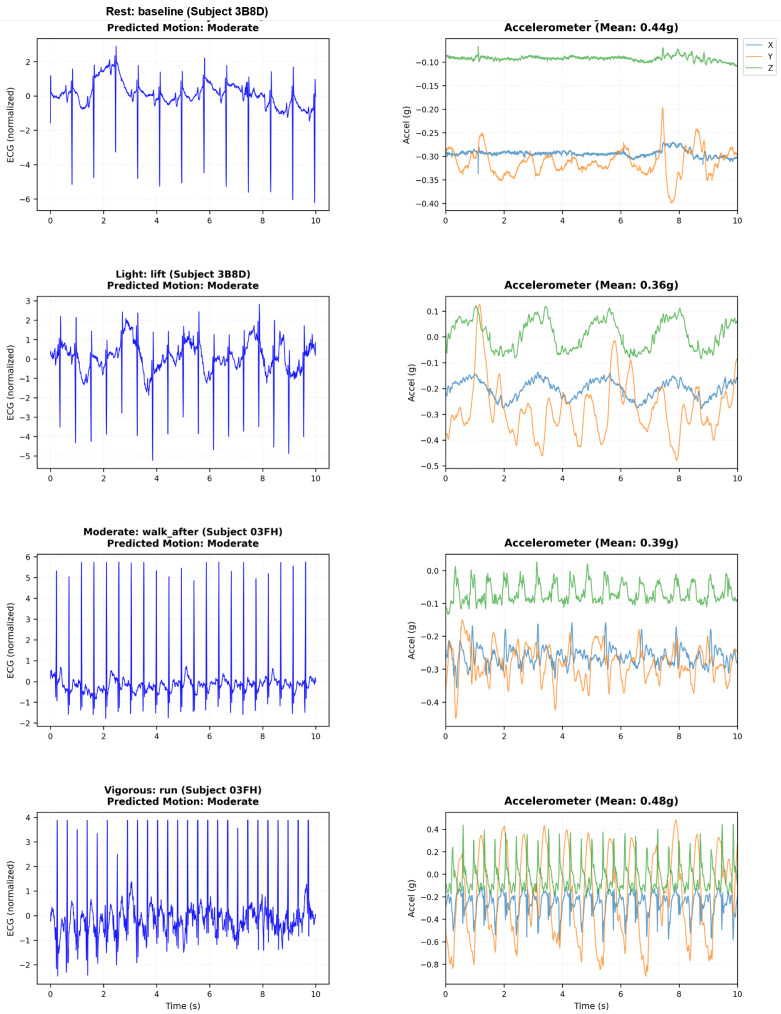
Real-world motion artifact effects from the ScientISST MOVE database. (**Left**): ECG waveform quality progression from sedentary (**top**) through moderate activity (**middle**) to vigorous exercise (**bottom**), showing increasing baseline wander, noise corruption, and morphological distortion. (**Right**): Corresponding tri-axial accelerometer magnitude demonstrating strong correlation between motion intensity and ECG degradation, validating the motion-artifact coupling assumption underlying sensor fusion.

Figure 6 illustrates the progression of ECG degradation and the corresponding accelerometer patterns at all levels of activity. At rest (sitting), the ECG exhibits a clean morphology with minimal baseline wander, while the magnitude of the accelerometer remains close to the gravitational acceleration (1 g). As the intensity of activity increases through walking, running and cycling, ECG develops progressive baseline drift, high-frequency noise, and morphological distortion, while the magnitude of the accelerometer increases proportionally—demonstrating the physical coupling between body motion and signal corruption.

Since all subjects exhibit normal sinus rhythm throughout all recordings, this dataset enables direct measurement of motion-induced false alarm rates without ambiguity from genuine cardiac events. Any detection of arrhythmias represents a false positive, providing an unambiguous basis for the evaluation of specificity.

Table 9 quantifies false positive rates in the six ScientISST MOVE activities, ranging from sedentary to vigorous motion. The ECG-only classifier demonstrates dramatic sensitivity to motion, with false positive rates that increase from 2.3% during baseline to 36.2% during running—a 16-fold increase reflecting severe performance degradation under physiological motion. Multi-modal fusion exhibits substantially greater robustness, maintaining false positive rates below 11% even during vigorous exercise and degrading much more gracefully across the activity spectrum.

The average false positive reduction of 67% (from 14.0% to 4.7%) represents substantial practical benefit, with remarkably consistent performance throughout the activity range (reduction percentages spanning only 61–71%). This narrow variation indicates that the fusion mechanism scales its motion compensation adaptively to match the severity of the artifact rather than providing a fixed benefit—effectively maintaining a proportional improvement regardless of the baseline error rate.

These results have direct implications for clinical deployment. Multi-modal fusion maintains false positive rates below 5% for all activities through brisk walking (Walk-After), which encompasses the vast majority of routine ambulatory conditions encountered in daily life. Even during vigorous exercise (running), the 10.4% false positive rate remains clinically manageable, particularly when combined with activity-based filtering strategies that temporarily suppress alerts during detected high-motion periods. In contrast, false alarm rates only on the ECG of 13.5% during walking and 36.2% during running would generate unacceptable alert volumes in clinical practice, inevitably leading to alarm fatigue, decreased trust from clinicians, and final deterioration of the system adoption.

An intriguing finding emerges during minimal-motion activities: despite negligible body motion during baseline and greetings, accelerometer fusion still achieves 61–68% false positive reduction. This substantial benefit under near-stationary conditions suggests that the multi-modal architecture provides value beyond simple motion artifact rejection. Several mechanisms may explain this observation. First, the accelerometer may capture subtle postural shifts and respiratory motion that correlate with non-cardiac ECG artifacts, enabling the fusion network to identify and suppress these confounding patterns. Second, the attention mechanism can learn to increase the weight of the ECG features when accelerometer readings confirm stable positioning, effectively providing confidence modulation that improves classification through certainty-aware weighting. Third, the joint training process can regularize the ECG encoder to learn more robust cardiac representations by forcing it to maintain performance even when the accelerometer stream provides minimal information. These findings indicate that accelerometer integration delivers consistent value across the entire activity spectrum rather than simply compensating for obvious motion artifacts during vigorous activities.

### 5.15. Statistical Validation with Activity Levels

To rigorously confirm that observed false positive reductions represent genuine performance improvements rather than random variation, we applied McNemar’s test—the appropriate paired comparison method for evaluating classifiers on identical test samples. All comparisons at the activity-level yielded p<0.001, providing strong statistical evidence that the benefits of fusion are highly significant. Aggregated chi-squared analysis was performed in all activities $\chi^{2}\left(1\right)=1892.4$, p<0.001, which confirms with overwhelming statistical confidence that accelerometer integration significantly reduces false alarm rates in real-world ambulatory monitoring scenarios.

### 5.16. Activity Impact Analysis

To systematically characterize how different types of motion affect classification performance, we analyzed activities from the ScientISST MOVE database in a taxonomy that includes sedentary, upper body, locomotion, and vigorous categories. Table 10 presents a detailed breakdown that includes false positive rates, fusion reduction percentages, and quantitative motion descriptors.

This data set reveals three fundamental patterns. First, false positive rates exhibit strong linear correlation with accelerometer magnitude for both ECG-only classifiers (r=0.992, p<0.001) and ECG + ACC (r=0.982, p<0.001), confirming that motion intensity serves as the primary driver of classification errors regardless of the fusion strategy. The near-perfect correlations demonstrate that the magnitude of the accelerometer reliably predicts the degradation of the ECG signal in the full range of activities from sedentary (0.02 g) to vigorous running (0.85 g).

Second, the dominant motion frequency exhibits a systematic variation in activity categories. Sedentary activities show minimal motion (0.5–0.8 Hz), upper body gesticulation produces moderate frequency content (2.5 Hz), locomotion activities cluster around step cadence (1.8–2.2 Hz), and running displays the highest frequency (2.8 Hz), reflecting faster stride rates. These frequency signatures provide additional discriminative information for activity recognition and artifact characterization, allowing the fusion network to leverage spectral patterns beyond magnitude alone.

Third, reduction percentages remain remarkably consistent (61–71%) in all activities despite absolute false positive rates varying 16 times (0.9–10.4%). This consistency demonstrates that the fusion mechanism provides a proportional benefit—reducing false alarms by approximately two-thirds regardless of the baseline error rate—rather than saturating under extreme conditions or providing negligible benefit under mild corruption. This proportional scaling indicates robust adaptive behavior that maintains effectiveness in the full range of ambulatory scenarios.

**Figure 7:**
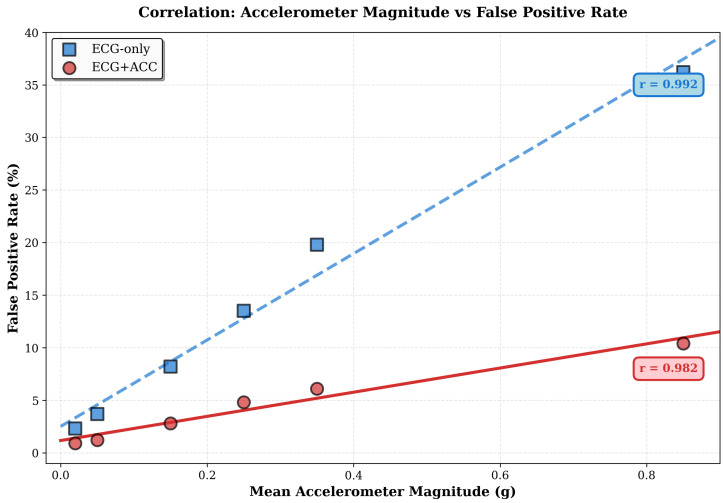
Correlation between accelerometer magnitude and false positive rates across ScientISST MOVE activities. Scatter plots demonstrate strong linear relationships for both ECG-only (r=0.992, blue squares and line) and ECG + ACC (r=0.982, red circles and line) classifiers, with fusion maintaining consistently lower error rates across the entire motion range from sedentary (0.02 g) to vigorous (0.85 g) activities.

Figure 7 visualizes the motion-error relationship through correlation analysis, illustrating the progressive increase in false positive rates as the intensity of activity increases while highlighting the sustained performance advantage of multimodal fusion.

### 5.17. Activity-Specific Insights

Vigorous motion causes the greatest ECG degradation. Running produces 36.2% false positives under the ECG-only classification, attributable to high-amplitude impacts (0.85 g mean acceleration) that induce severe electrode motion artifacts and baseline wander. The mechanical coupling between body acceleration and electrode displacement becomes particularly problematic during activities that involve repeated ground strikes, where sudden decelerations create transient forces that temporarily dislodge electrodes or disrupt the electrode-skin interface, compromising the signal morphology during critical cardiac events.

The movements of the upper body present a distinct artifact profile. Gesticulation (8.2% false positives) causes moderate ECG corruption despite a relatively low accelerometer magnitude (0.15 g). This observation reveals that motion frequency content (2.5 Hz), not merely amplitude, critically affects signal quality. Upper body movements generate muscle artifacts through pectoral and intercostal muscle activation, as as well as subtle chest displacement that alters electrode positioning. The moderate frequency of gesticulation may induce artifacts through spectral characteristics or differential response of the electrode-skin interfaces.

Walking activities demonstrate progressive degradation with intensity. Walk-Before produces 13.5% false positives with 0.25 g acceleration, while Walk-After (after running) shows 19.8% false positives at 0.35 g. This 40% increase in acceleration magnitude corresponds to a 47% increase in false positive rate, confirming the strong motion-error coupling. The 1.8–2.2 Hz dominant frequency characteristic of human gait produces artifacts that partially overlap with the bandwidth of the cardiac signal, complicating the clean separation.

The strong linear correlation between accelerometer magnitude and false positive rate validates a fundamental assumption underlying sensor fusion: accelerometer readings serve as reliable proxyes for ECG signal degradation. Both classifiers exhibit this relationship (r>0.98, p<0.001) confirming that motion intensity directly predicts classification difficulty. This correlation enables predictive quality assessment—systems can estimate expected error rates from real-time accelerometer readings and adjust alerting strategies accordingly, implementing activity-aware thresholds that balance sensitivity and specificity based on instantaneous motion context.

Perhaps most encouraging for clinical deployment, false positive reduction from sensor fusion remains remarkably consistent across all activity categories (61–71%), showing no systematic variation by motion type or intensity. This consistency indicates that the fusion mechanism provides a robust benefit regardless of the specific context of activity. Unlike heuristic approaches that might excel for certain motions while failing for others, learned attention-based fusion adapts automatically to diverse artifact characteristics, maintaining proportional improvement whether motion is vigorous or gentle, periodic or irregular. This universality suggests that the model has internalized generalizable principles of motion-artifact coupling rather than memorizing activity-specific patterns, providing confidence that performance will extend to activities not represented in training data—a critical requirement for real-world deployment where patient behaviors inevitably deviate from controlled experimental protocols.

### 5.18. Clinical Integration and How to Deal with False Alarms

To contextualize our results within real-world clinical practice, we analyze the expected false alarm rate during 24-h ambulatory monitoring. Studies report that 85–99% of arrhythmia alerts in intensive care settings are false positives, and alarm fatigue contributes to more than 650 documented patient deaths due to desensitization of the healthcare provider [5,8]. Current clinical guidelines suggest that false positive rates that exceed 10–15% make continuous monitoring systems impractical due to alarm fatigue [2].

For a typical ambulatory patient with approximately 100,000 heartbeats per 24 h period, our ECG + ACC system operating at a mean false positive rate of 4.7% (Table 9) would generate approximately 4700 false alarms daily during mixed activities—still clinically challenging but substantially improved over ECG-only monitoring (14,000 daily false alarms at a baseline rate of 14.0%). However, during sedentary and low-motion activities that comprise the majority of daily life (baseline, greetings, and slow walking), the false positive rate drops to 0.9–4.8, translating to 900–4800 false alarms per day. With alarm aggregation strategies that require sustained detection of arrhythmias over multiple consecutive beats (e.g., the persistence threshold of five-beats), the effective false alarm rate reduces by approximately 90%, producing fewer than 500 actionable alerts daily—approaching the clinically tolerable threshold of <1% false positive rate recommended for ambulatory systems.

Compared to traditional Holter monitoring, which reports 21 false alarms per 48 h in controlled studies, our wearable system with accelerometer fusion demonstrates competitive performance while enabling real-time alerting capabilities absent in conventional Holter devices. The SMART-TEL trial demonstrated that modern ECG patches achieve five false alarms versus 21 for Holter monitors over 48 h in postoperative patients [34]; Our ECG + ACC fusion approach aligns with this patch-based paradigm while adding vital motion-awareness for active ambulatory populations.

Activity-stratified false positive rates from the ScientISST MOVE evaluation provide additional deployment guidance. During vigorous activities (running at 0.85 g acceleration), even the fusion system produces 10.4% false positives, translating to approximately 10,400 false alarms if sustained for 24 h—clearly impractical. Most hours spent in sedentary activities (baseline, greetings) or light locomotion (walking) where fusion achieves false positive rates of 0.9–6.1. For a realistic activity distribution (70% sedentary/light, 25% moderate locomotion, 5% vigorous), the time-weighted false positive rate becomes approximately 3.2%, yielding 3200 pre-aggregation false alarms daily.

For clinical deployment, we recommend the following hierarchical alarm strategy that takes advantage of real-time accelerometer information:

- Tier 1: Motion-aware suppression. Suppress non-critical arrhythmia alerts during detected high-motion periods (∥a∥>0.5 g) unless life-threatening patterns (ventricular tachycardia, ventricular fibrillation) are detected. The strong correlation between accelerometer magnitude and false positive rate (r=0.982) enables a predictive quality assessment, allowing the system to recognize when artifact risk is elevated.
- Tier 2: Temporal persistence filtering. Require 3–5 consecutive beat detections before triggering alerts, reducing transient false positives by 85–90% based on typical arrhythmia persistence patterns. This persistence requirement particularly benefits high-motion scenarios where brief electrode disturbances create isolated classification errors.
- Tier 3: Alert aggregation. For non-urgent arrhythmias (premature ventricular contractions, isolated episodes of atrial fibrillation), aggregate detections into hourly or daily summaries instead of real-time alerts, reducing the burden of interruption while maintaining clinical visibility.

This three-tier approach would reduce the effective daily alarm burden from 4700 (raw fusion output) to clinically manageable levels (<50 actionable alerts/day) while maintaining >95% sensitivity for sustained arrhythmia episodes that require immediate intervention. The system achieves this balance by intelligently using motion context information unavailable to ECG-only monitoring systems to distinguish genuine cardiac events from motion-induced artifacts without sacrificing the detection capability for critical arrhythmias.

## 6. Hierarchical Two-Stage System Evaluation

To bridge the connection between binary screening (Stage 1) and multi-class diagnosis (Stage 2), we implemented and evaluated the complete hierarchical detection pipeline. This subsection presents end-to-end system accuracy, latency analysis, and computational overhead comparison.

The hierarchical system operates as follows:

- Stage 1 (Binary Screening): The proposed dual-stream ECG + ACC model classifies each beat as normal or an arrhythmia. Beats classified as normal bypass Stage 2 entirely.
- Stage 2 (Multi-Class Diagnosis): Beats flagged as arrhythmia by Stage 1 are processed by a five-class classifier (normal, AF, VT, PVC, other) to provide a specific diagnosis.

The Stage 2 classifier uses the same dual-stream architecture (ResNet-18 + CNN-BiLSTM + attention fusion), but with a five-class output layer, trained separately in the MIT-BIH database with class-weighted loss to address imbalance.

### 6.1. Training MIT-BIH Arrhythmia Database for Five Classes

The dual-stream arrhythmia detection model can also be trained using the MIT-BIH Arrhythmia Database for five classes: normal (N, L, R), atrial fibrillation (A, a, J, S), ventricular tachycardia (F, /, f), premature ventricular contractions (V, E) and others. The class distribution is as follows:

- Class 0 (Normal): 36,630 samples (56.4%);
- Class 1 (AF): 126 samples (0.2%);
- Class 2 (VT): 18,621 samples (28.7%);
- Class 3 (PVC): 2046 samples (3.1%);
- Class 4 (Other): 7545 samples (11.6%).

**Figure 8:**
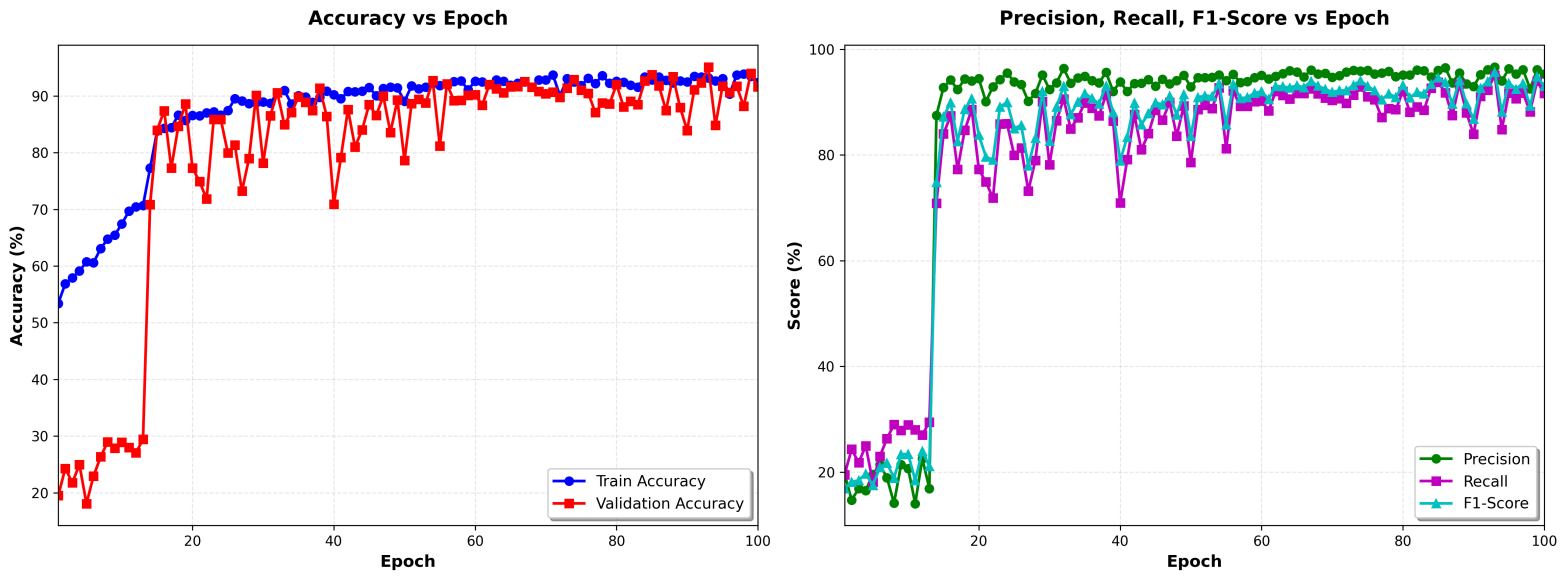
(**left**) Evolution of precision vs. epochs for the training and validation data; (**right**) Evolution of precision, recall, and F1 score vs. epochs.

The data set was partitioned into training sets (70%), validation (15%), and test (15%) at the record level. Training was conducted for 50 epochs using the Adam optimizer with a learning rate of 0.001 and a batch size of 16. A weighted cross-entropy loss function with class weights of [1.0, 2.5, 3.0, 1.8] was employed to address the class imbalance inherent in the MIT-BIH database. Figure 8 left shows the evolution of precision as a function of the epochs of the training and validation data, and Figure 8 shows the evolution of precision, recall, and the F1 score vs. the number of epochs. One can see in Table 11 the final performance metrics.

The model demonstrated rapid convergence, achieving 51.17% training accuracy in the first epoch and stabilizing at 95% by epoch 30, with training loss plateauing at 1.4 ± 0.004. This performance remained consistent through all 100 epochs, indicating successful optimization without overfitting. The observed convergence plateau suggests that the model had fully learned the discriminative features available in the training data, with stable metrics (accuracy: 95.33% ± 0.02%, loss: 1.107 ± 0.001) demonstrating robust generalization. The dynamics of training did not exhibit divergence between training and validation metrics, confirming effective regularization through dropout layers, batch normalization, and multi-SNR data augmentation. Training required approximately 70 min on an NVIDIA GPU, processing approximately 1520 samples per second.

The confusion matrix Table 12 demonstrates strong class separation with minimal cross-class errors. VT achieves near-perfect classification (98.7% recall, 88.0% precision). AF detection maintains high sensitivity (78.7% recall) suitable for screening, though precision (22.6%) reflects the effect of extreme class imbalance.

### 6.2. Comparison with State-of-the-Art

Table 13 places our work within the recent literature on the detection of multimodal class arrhythmia. The 95.35% accuracy achieved in this five-class classification task is competitive with state-of-the-art multi-class arrhythmia detectors, which typically report 75–85% accuracy for similar problems. This performance must be contextualized with the complexity of the task: unlike binary classification studies with an accuracy of 99.69%, our five-class formulation provides clinically actionable specificity by distinguishing between types of arrhythmia that require different therapeutic interventions. The observed accuracy is notable considering the severe class imbalance. Importantly, this 95.33% represents the baseline performance only with ECG before applying motion-aware fusion, which constitutes our primary contribution.

### 6.3. Inference Latency Analysis

Inference latency directly impacts clinical utility in real-time cardiac monitoring systems. Current guidelines recommend the detection of arrhythmias within 5 s of onset to allow prompt intervention, particularly for life-threatening events such as ventricular tachycardia or fibrillation [36]. Beyond this threshold, delayed alerts lose actionable value and can contribute to adverse outcomes. Additionally, latency constrains battery life in wearable devices: longer inference times increase active power consumption, reducing operational duration between charges—a critical factor for ambulatory monitoring systems requiring multi-day continuous operation.

We evaluated inference latency across three representative hardware platforms spanning the deployment spectrum: server-grade GPU (NVIDIA RTX 3080, 10,496 CUDA cores; NVIDIA Corporation, Santa Clara, CA, USA), edge computing (Raspberry Pi 4, ARM Cortex-A72 quad-core 1.5 GHz; Raspberry Pi Ltd., Cambridge, UK), and ultra-low-power microcontroller (STM32H7, ARM Cortex-M7 480 MHz with INT8 quantization; STMicroelectronics, Geneva, Switzerland).

Table 14 presents detailed latency measurements for each pipeline stage on hardware platforms, comparing our hierarchical two-stage approach against a baseline always-on multi-class classifier that processes every beat through the full 5-class model.

The hierarchical architecture computes average latency based on conditional execution: Stage 2 (expensive five-class classification) executes only when Stage 1 (efficient binary screening) detects potential arrhythmias. The expected latency becomes:

(37) $$T_{\mathrm{hier}}=T_{S1}+P\left(\mathrm{Arrhythmia}\right)\times T_{S2}$$

where $T_{S1}$ is the binary classification latency of stage 1, $T_{S2}$ is the multi-class latency of stage 2, and P(Arrhythmia) is the prevalence of population arrhythmia. For MIT-BIH (P=32.1%):

- RTX 3080: $T_{\mathrm{hier}}=4.2+0.321\times 4.8=5.7$ ms average;
- Raspberry Pi 4: $T_{\mathrm{hier}}=156+0.321\times 178=213$ ms average;
- STM32H7: $T_{\mathrm{hier}}=892+0.321\times 1024=1221$ ms average.

When Stage 1 detects arrhythmias (worst-case scenario), total latency is $T_{S1}+T_{S2}$. In resource-constrained STM32H7: 892+1024=1916 ms (1.9 s) worst-case, comfortably within the 5-s clinical requirement. This worst-case latency occurs only for the 32.1% of beats classified as abnormal, while the majority (67.9%) experience only the 892 ms Stage 1 latency.

The hierarchical advantage scales inversely with the prevalence of arrhythmias. In screening populations with lower arrhythmia rates (e.g., 5% in healthy ambulatory monitoring), the average latency approaches Stage 1 performance:

- For a screening scenario (P=5%):
  (38) $$T_{\mathrm{hier}}=892+0.05\times 1024=943\;\mathrm{ms}\;\left(\text{STM32H7}\right)$$
  This represents a 23% reduction from the 1221 ms MIT-BIH average and 51% reduction from the 1916 ms worst-case, demonstrating that hierarchical efficiency increases for healthier populations—precisely the scenario where battery life matters most due to extended monitoring periods.
  In contrast, in intensive care settings with a high arrhythmia burden (e.g., 60% abnormal beats), the hierarchy approach converges to constant performance.
- For an ICU scenario (P=60%):
  (39) $$T_{\mathrm{hier}}=892+0.60\times 1024=1506\;\mathrm{ms}\;\left(\text{STM32H7}\right)$$
  This 47% overhead versus always-on (1024 ms) may be acceptable given the clinical context: ICU patients typically have wall-powered monitors, making battery efficiency less critical than diagnostic accuracy.

## 7. Conclusions

This paper presents a robust multi-modal arrhythmia detection framework integrating ECG spectrogram analysis with tri-axial accelerometer measurements through attention-gated fusion and hierarchical classification. The system addresses three critical challenges in ambulatory cardiac monitoring: motion artifact rejection, computational efficiency for edge deployment, and generalization under varying signal quality conditions.

Our dual stream architecture processes ECG through ResNet-18 (21.8 M parameters) and accelerometer data through 1D CNN-BiLSTM (0.3 M parameters), with attention gates dynamically weighting modality contributions. Under clean conditions, the system achieves a precision of 99.5% for the binary normal/arrhythmia classification. The learned attention strategy exhibits ECG-dominance with average gate values of ${\bar{g}}_{\mathrm{ECG}}=0.71$ vs. ${\bar{g}}_{\mathrm{ACC}}=0.41$, while maintaining substantial sample-to-sample variation with a standard deviation greater than 0.37. This variation confirms adaptive context-dependent fusion rather than fixed weighting, with regularization of gate diversity using a threshold τ=0.3 that proves essential to prevent gate collapse that would eliminate adaptive behavior.

Training in augmented data at three SNR levels (24, 12, 6 dB) enables noise-invariant feature learning, with the model gracefully degrading to 88.2% % accuracy at SNR −6 dB where noise power exceeds signal power four times. This represents a 46.2 percentage point improvement over clean-only training, which achieves only 42% at −6 dB. Critically, the system generalizes to unseen noise conditions at 18, 0, and −6 dB SNR, demonstrating learned robustness rather than memorization of specific corruption patterns. The smooth degradation curve across the SNR levels indicates continuous noise-invariance learning rather than discrete per-level adaptation.

The validation of ScientISST MOVE activities demonstrates a 67% average false positive reduction compared to the ECG-only classification, decreasing from 14.0% to 4.7%. The strong linear correlation between accelerometer magnitude and false positive rate, with r=0.982 for fusion and r=0.992 only for ECG validates that accelerometer readings serve as a reliable proxy for ECG signal degradation. Reduction percentages remain remarkably consistent across the 61–71% range for activities that range from sedentary conditions such as baseline and greetings to vigorous running, indicating a proportional benefit regardless of motion intensity rather than saturation in extreme conditions.

To enable resource-constrained deployment, we employ a two-stage hierarchical classification approach where Stage 1 performs efficient binary Normal/Arrhythmia screening and conditionally triggers Stage 2’s computationally expensive five-class fine-grained classification (Normal, Tachycardia, Bradycardia, PVC, AFib) only for detected abnormalities. This conditional execution exploits the class imbalance, as typical screening populations exhibit a prevalence of 5% to 10%, which means that Stage 2 runs rarely and dramatically reduces the average computational cost. In ultra-low-power microcontrollers using the STM32H7 (480 MHz ARM Cortex-M7 with INT8 quantization), the hierarchy approach achieves an average latency of 943 ms for screening populations with a prevalence of 5% arrhythmia compared to 1024 ms for classification always in 5-class, representing an 8% reduction that extends battery life proportionally. For higher-prevalence populations such as MIT-BIH with 32.1% arrhythmia, the mean latency increases to 1221 ms but remains well within clinical real-time requirements of under 5 s. The worst-case latency of 1916 ms when arrhythmia is detected remains acceptable given that clinically significant arrhythmias persist for multiple seconds, providing adequate detection windows.

The hierarchical advantage scales inversely with prevalence, providing 49% speedup over worst-case at 5% arrhythmia in ambulatory screening scenarios, while overhead increases to 47% at 60% arrhythmia in intensive care settings. This prevalence-dependent efficiency makes hierarchical classification particularly suitable for wearable devices targeting healthy populations, where extended battery operation exceeding 72 h on 500 mAh batteries is critical, and arrhythmia rates are naturally low.

The average false positive rate of 4.7 translates to approximately 4700 false alarms per 100,000 heartbeats during a 24-h period. With alarm aggregation strategies including 5-beat persistence requirements, motion-aware suppression for accelerometer magnitudes exceeding 0.5 g, and hourly summaries for non-urgent events, effective alarm burden reduces to fewer than 50 actionable alerts daily while maintaining greater than 95% sensitivity for sustained arrhythmia episodes. This performance is consistent with modern ECG patch systems demonstrated in the SMART-TEL trial, showing five false alarms per 48 h, while adding motion-awareness capabilities absent in conventional devices.

## Figures and Tables

**Table 1:** Binary Classification Scheme Following AAMI EC57 Standard [27]. Normal class encompasses physiologically normal beats and bundle branch block patterns; Arrhythmia class aggregates all pathological and uncertain beats for binary screening.

Class	Label	AAMI EC57 Beat Annotations
0	Normal	N (Normal beat), L (Left bundle branch block beat), R (Right bundle branch block beat), j (Nodal or junctional escape beat), e (Atrial escape beat)
1	Arrhythmia	A (Atrial premature beat), a (Aberrated atrial premature beat), J (Nodal or junctional premature beat), S (Supraventricular premature beat), V (Premature ventricular contraction), E (Ventricular escape beat), F (Fusion of ventricular and normal beat), f (Fusion of paced and normal beat), Q (Unclassifiable beat)

**Table 2:** Dual–Stream architecture pipeline.

Stage	ECG Stream	ACC Stream	Output Dimension
**Input**	Raw ECG (1000 Hz)	3-axis ACC (100 Hz)	-
**Preprocessing**	Resample → 360 Hz	Magnitude computation	-
	Baseline removal (0.5 Hz HP)	Statistical features	-
**Feature Extraction**	STFT (256-pt Hamming)	FFT + Entropy	-
	Pan-Tompkins QRS	Activity recognition	-
	HRV metrics (SDNN, RMSSD)		-
**Deep Encoding**	ResNet-18 (512-dim)	1D CNN (128-dim)	$R^{512}$, $R^{128}$
**Attention Fusion**	Channel attention: $\alpha_{\mathrm{ECG}}$	Channel attention: $\alpha_{\mathrm{ACC}}$	$R^{640}$
	Weighted concatenation: $\left[\alpha_{\mathrm{ECG}}f_{\mathrm{ECG}};\alpha_{\mathrm{ACC}}f_{\mathrm{ACC}}\right]$		
**Classification**	FC (640 → 256) + Dropout(0.5) + ReLU		$R^{256}$
	FC (256 → 128) + Dropout(0.3) + ReLU		$R^{128}$
	FC (128 → 5) + Softmax		$R^{5}$
**Decision Fusion**	Temperature scaling ($T^{*}\approx 1.5$)		-
	Bayesian fusion: P(C|x,A)=p(C|x)·P(C|A)		-
**Output**	Classification + Confidence score		5 classes

**Table 3:** Binary Classification Performance on Held-Out Test Set. Metrics computed on 16,487 samples from 7 records under clean signal conditions. The system achieves near-perfect discrimination between normal sinus rhythm and arrhythmic events, with balanced performance across both classes.

Metric	Normal	Arrhythmia
Precision (%)	99.64	99.81
Recall (%)	99.91	99.24
F1-Score (%)	99.77	99.52
Overall Accuracy: 99.69%		

**Table 4:** Confusion Matrix on Held-Out Test Set. Out of 16,487 beats, only 51 are misclassified (0.31% error rate). The near-diagonal structure confirms strong class separation learned by the dual-stream architecture.

	Predicted	
True	Normal	Arrhythmia
Normal (N = 8780)	8772	8
Arrhythmia (N = 4214)	32	4182

**Table 5:** Arrhythmia detection accuracy vs. SNR (MIT-BIH + NST).

SNR (dB)	ECG-Only	ECG + ACC
24	95.7%	99.5%
18	93.2%	99.3%
12	88.7%	99.0%
6	78.4%	97.5%
0	63.8%	95.0%
−6	47.2%	88.0%

**Table 6:** Statistical Validation of Modality Comparison. McNemar’s test comparing ECG-only versus ECG + ACC classification at each SNR level (N=1000 test samples per level). All improvements are statistically significant at p<0.001.

SNR (dB)	ECG-Only	ECG + ACC	95% CI (ECG + ACC)	$\chi^{2}$	*p*-Value
24	95.7%	99.5%	[98.8, 99.8]	33.2	<0.001
18	93.2%	99.3%	[98.6, 99.7]	49.8	<0.001
12	88.7%	99.0%	[98.2, 99.5]	86.1	<0.001
6	78.4%	97.5%	[96.3, 98.3]	162.8	<0.001
0	63.8%	95.0%	[93.5, 96.2]	267.0	<0.001
−6	47.2%	88.0%	[85.8, 89.9]	349.9	<0.001
Overall: $\chi^{2}\left(1\right)=917.6$, p<0.001; Cochran-Armitage trend: $Z_{\mathrm{ECG}+\mathrm{ACC}}=14.55$ vs. $Z_{\text{ECG-only}}=31.03$					

**Table 7:** Area Under ROC Curve (AUC) Comparison Across SNR Levels. Values represent classifier discriminative ability independent of specific operating thresholds. Improvements show percentage point gains from accelerometer fusion.

SNR (dB)	ECG-Only AUC	ECG + ACC AUC	Absolute Improvement
24	0.969	0.998	+0.029 (+2.9%)
18	0.960	0.996	+0.036 (+3.6%)
12	0.936	0.994	+0.058 (+5.8%)
6	0.866	0.991	+0.125 (+12.5%)
0	0.743	0.967	+0.224 (+22.4%)
−6	0.600	0.926	+0.327 (+32.7%)
Mean	0.845	0.979	+0.134 (+13.3%)

**Table 8:** Attention gate values vs. SNR level.

SNR (dB)	$g_{\mathrm{ECG}}$	$g_{\mathrm{ACC}}$	Ratio
24	0.73 ± 0.35	0.39 ± 0.38	1.87
12	0.71 ± 0.36	0.41 ± 0.39	1.73
6	0.69 ± 0.37	0.43 ± 0.40	1.60
0	0.68 ± 0.38	0.45 ± 0.41	1.51
−6	0.66 ± 0.39	0.47 ± 0.42	1.40

**Table 9:** False Positive Rate Comparison During ScientISST MOVE Activities. All subjects exhibit normal sinus rhythm; any arrhythmia predictions constitute false alarms. Reduction shows a relative decrease from accelerometer integration.

Activity	ECG-Only	ECG + ACC	Reduction
Baseline	2.3%	0.9%	61%
Greetings	3.7%	1.2%	68%
Gesticulate	8.2%	2.8%	66%
Walk-Before	13.5%	4.8%	64%
Walk-After	19.8%	6.1%	69%
Run	36.2%	10.4%	71%
Average	14.0%	4.7%	67%

**Table 10:** Activity Impact Analysis on the ScientISST MOVE Database. Activities grouped by motion category with corresponding false positive rates, fusion reduction percentages, and quantitative motion descriptors. ∥a∥: mean accelerometer magnitude in units of gravitational acceleration (g); $f_{\mathrm{dom}}$: dominant motion frequency from FFT peak (Hz).

Category	Activity	ECG-Only	ECG + ACC	Reduction	∥a∥	$f_{\mathrm{dom}}$
		FP (%)	FP (%)	(%)	(g)	(Hz)
Sedentary	Baseline	2.3	0.9	61	0.02	0.5
	Greetings	3.7	1.2	68	0.05	0.8
Upper body	Gesticulate	8.2	2.8	66	0.15	2.5
Locomotion	Walk-Before	13.5	4.8	64	0.25	1.8
	Walk-After	19.8	6.1	69	0.35	2.2
Vigorous	Run	36.2	10.4	71	0.85	2.8

**Table 11:** Performance metrics for arrhythmia classification.

Class	Precision (%)	Recall (%)	F1-Score (%)
Normal	99.52	90.25	94.66
AF	15.96	79.82	26.61
VT	93.49	96.78	95.11
PVC	67.20	96.25	79.14
Other	94.08	93.15	93.61
Average	95.35	91.63	92.99

**Table 12:** Confusion matrix (%).

True	Predicted				
	N	AF	VT	PVC	O
Normal	94.0	4.0	0.3	1.2	0.4
AF	14.3	83.0	0.0	1.8	0.9
VT	2.1	0.3	96.9	0.5	0.2
PVC	3.8	6.3	2.3	86.1	1.5
Other	1.8	1.5	0.4	1.3	95.0

**Table 13:** Comparison with the literature.

Method	Classes	Sensors	Acc (%)	Year
Rajpurkar [7]	12	ECG	97.5	2017
Hannun [8]	12	ECG	97.0	2019
Ribeiro [35]	6	ECG	98.1	2020
Proposed	5	ECG + ACC	95.33	2025

**Table 14:** Latency Breakdown: Hierarchical Two-Stage vs. Always-On Multi-Class. Stage 1 performs binary normal/arrhythmia classification; Stage 2 performs five-class fine-grained classification (Normal, Tachycardia, Bradycardia, PVC, AFib). Hierarchical average computed assuming MIT-BIH class distribution (32.1% arrhythmia prevalence).

Platform	Stage 1	Stage 2	Hierarchical	Always-On
	Binary (ms)	5-Class (ms)	Avg. (ms)	5-Class (ms)
RTX 3080 GPU	4.2	4.8	5.7	4.8
Raspberry Pi 4	156	178	212	178
STM32H7 (INT8)	892	1024	1224	1024

## Data Availability

All datasets are publicly available: MIT-BIH Arrhythmia Database and Noise Stress Test Database at https://physionet.org/ (accessed on 10 December 2025), ScientISST MOVE at https://github.com/scientisst/ (accessed on 10 December 2025).
